# Spatiotemporal dynamics of heat stress and cold stress on UK rapeseed cropping over 1961–2020

**DOI:** 10.1038/s41598-026-41957-7

**Published:** 2026-03-05

**Authors:** Biao Hu, Mark E. J. Cutler, Alexandra C. Morel

**Affiliations:** Division of Energy, Environment & Society, School of Humanities, Social Sciences and Law, University of Dundee, Nethergate, Dundee, DD1 4HN UK

**Keywords:** Rapeseed, Heat stress, Cold stress, Spatiotemporal analysis, Production loss index, Climate sciences, Ecology, Ecology, Environmental sciences, Plant sciences

## Abstract

Most temperature stress research in oil crops has focused on either heat or cold stress with analyses of the effects of both in combination rare. For the UK, neither the spatiotemporal hot spots of temperature stressed arable areas nor the comparative trends of heat and cold stresses for rapeseed cropping under climate change are understood. This study investigated the spatiotemporal heat and cold stresses for UK rapeseed over 1961–2020, and quantified the normalized rapeseed production loss index (*f*_*RPL*_) induced by heat stress during flowering. Stress indices including a literature derived heat stress index (*f*_*HS*_), cold degree days (*CDD*), with historical land cover and crop productivity data were used to comparatively study both stresses and to estimate *f*_*RPL*_. Results showed increasing *f*_*HS*_, particularly during flowering (April to May) and main yield-forming reproductive stages (spanning flowering through pod and seed development from April to July) over the study period, with *f*_*HS*_ being negatively correlated with latitude. The decreasing values of *CDD* and frequency of cold stress suggest cold stress decreased during the flowering, vegetative (September to November) and reproductive stages. Notably, this study observed that annually at the UK level heat stress was increasing at a faster rate than cold stress was decreasing during flowering. The increasing values of *f*_*RPL*_, with significant differences between decades and regions, suggested an increasing production loss. These results presented a potentially trend of increasing heat stress impacts on future rapeseed production and further work is required to understand the specific impacts and mitigation strategies for addressing UK food security.

**Supplementary Information:**

The online version contains supplementary material available at 10.1038/s41598-026-41957-7.

## Introduction

The success of agricultural activities is subject to many environmental factors. For instance, while temperature is one of significant determinants of agricultural production, heat stress can reduce crop yields if temperatures exceed critical high temperatures (*T*_*cri_h*_) or even limit/ceiling high temperatures (*T*_*limit_h*_) of crops^1–4^. Similarly, noticeable yield damage can be caused by cold stress when daytime temperatures drop below critical low temperatures (*T*_*cri_c*_)^5–7^. However, most research on temperature stress of agricultural crops, especially oil crops, has focused on either heat or cold stress in isolation, with few exploring the combined effects of both on crop production^1,6–10^. Together, these studies highlight the importance of temperature extremes for crop productivity, but also reveal that the combined effects of heat and cold stress on crops remain insufficiently explored. In addition to temperature, rainfall and water availability are also key determinants of agricultural production, particularly for rainfed cropping systems such as rapeseed^11–14^. Previous studies have shown that drought and water deficit can constrain rapeseed growth and yield, either independently or through interactions with temperature stress^15–17^. Nevertheless, temperature extremes during sensitive growth stages can impose direct physiological constraints on crop development that are not fully captured by rainfall metrics alone, justifying a focused assessment of temperature stress.

Although crop production is influenced by multiple climatic factors, few studies have jointly examined heat and cold stress in rapeseed. Among the exceptions, a global analysis of projected rapeseed production for 2040–2059 considered both heat and cold stress for RCP 8.5 and found that heat stress is expected to increase and cold stress will decrease compared to current conditions^18^. Despite this example, investigations relevant to aggregated heat and cold stresses currently being experienced and their spatiotemporal changes on oil crops are rare. In addition, with the spatially heterogeneous influence of global warming on surface temperatures, the distributions of crop impacts from temperature stresses are expected to vary by region^19–21^. Considering the importance for understanding crop stresses and their adaptation to climate change, more research activities for this field across varying geographical regions are needed.

Rapeseed is the primary oilseed (Fig. S1a) cultivated by area in the UK, where the winter type dominates^22^. The British rapeseed crop, which is mainly *Brassica napus* (*B. napus*) has nine principal growth stages (Fig. S2) based on the *Biologische Bundesanstalt*,* Bundessortenamt und CHemical Industry* (BBCH) system (https://ahdb.org.uk/knowledge-library/the-growth-stages-of-oilseed-rape)^23–25^. The oilseed is primarily used for edible oil, and increasingly as a feedstock for biofuels, highlighting its importance for national food security and sustainable energy supply^26,27^. Temperature is one of the determinants of rapeseed growth and development^9^. The temperature requirements and sensitivity of rapeseed to temperature differences can differ between growth stages^8,24^. Yield loss due to heat stress can be reflected by a decline of the performance of yield components including the number of flowers, number of pods per plant, number of seeds per pod and seed weight^28,29^. These studies indicate that rapeseed yield components are highly sensitive to temperature variability during reproductive development, suggesting that both high- and low-temperature stresses during flowering may be critical determinants of final yield. Among the studies focusing on UK rapeseed, some have investigated temperature variations on plant development. For instance, some researchers have studied the impacts of temperatures on germination, and found in some cases a non-linear response and others a linear response with increasing temperatures^30,31^. Additionally, by analysing aggregated national on-farm yield data and UK Agriculture and Horticulture Development Board (AHDB) annual Recommended List variety trial data, Brown and coworkers found that yield instability of UK’s winter rapeseed is significantly modulated by early winter (e.g. 27 November-21 December) temperatures^32^. Lu and colleagues revealed that warming during the period from middle autumn to early winter can also impact crop development (vernalization, dormancy, flowering time, etc.) and final yield of UK rapeseed^33^. Collectively, these studies demonstrate the sensitivity of UK rapeseed systems to temperature variability, but they predominantly focus on specific seasons, processes, or stress types rather than jointly considering heat and cold stress across space and time.

Whilst the impacts of heat and cold stresses on the flowering period of rapeseed have commonly been explored individually and the impacts of varying temperatures’ on UK rapeseed noted, little work has been done to quantify spatiotemporal variation in both stresses during flowering of rapeseed across different UK regions^6,29–38^. Additionally, while global distributions of heat stress on four crops including soybean for the periods 1971–2100 have been reported, peer reviewed quantitative frameworks identifying hotspots of temperature stress for UK rapeseed are not available^1^. Addressing these gaps is essential for understanding climate impacts on the UK’s primary oilseed crop and assessing emerging regional risk patterns.

Our study builds upon the work of Teixeira and colleagues, who studied heat stress for a thermally sensitive 30-day period (primarily the reproductive phases) of four crops^1^. As noted by the authors, one of the implications of their study might be an underestimation (given the whole of the flowering period to be sensitive) of heat stress. In rapeseed, sensitivity to temperature is not limited to heat stress, the crop is also sensitive to cold stress. Exposure to cold stress (7/0°C for day/night) during flowering has also been shown to damage pod filling rate and reduce final yield decreased by 12.23–18.5% despite compensatory increases in branching and flowering duration^6,29,37^. In maize, Li and coworkers demonstrated production losses during flowering when plants were exposed to cold stress (in chilling growing degree days)^39^. These previous results tended to suggest that more accurate estimation of temperature stress of rapeseed can be made if the whole of the flowering stage is considered and both the occurrence of heat and cold stress examined.

The present study therefore aimed to conduct a spatiotemporally explicit assessment of heat and cold stress on UK arable soils for rapeseed cropping, focusing on the temperature-sensitive flowering period, and to examine how these stresses and their impacts on rapeseed productivity have changed regionally and temporally from 1961 to 2020. We hypothesise that heat stress during the flowering period has intensified and cold stress declined over recent decades, but that relative importances and spatial distribution of these stresses vary across UK regions. By jointly analysing heat and cold stress across space and time, this study identifies regional hotspots and shifting temperature-risk profiles for UK rapeseed, providing new insight into how climate warming is reshaping the balance between opposing temperature stresses and highlighting where targeted adaptation strategies may be most urgently required.

## Materials and methods

### Study area and dataset

**Fig. 1:**
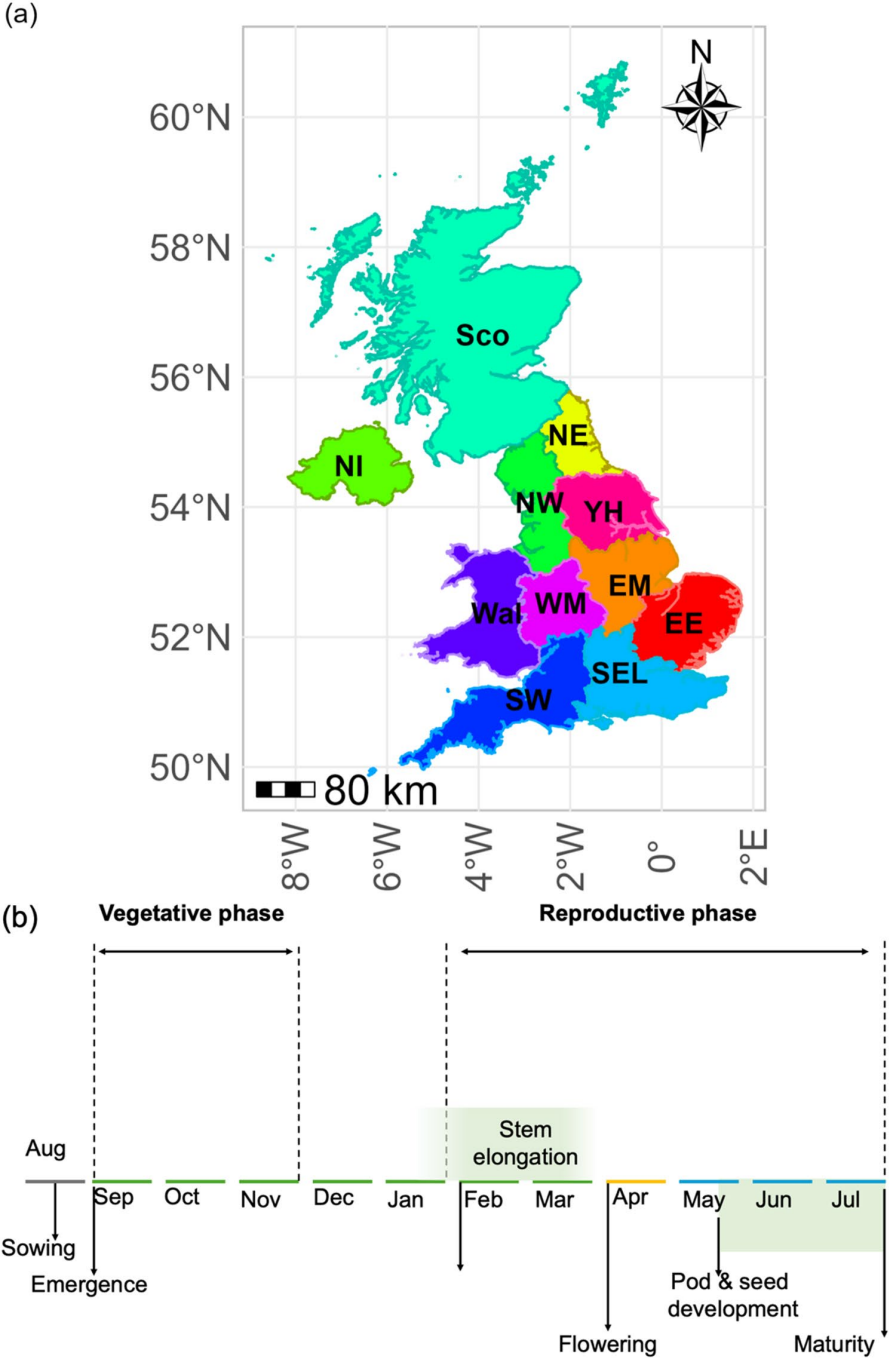
(**a**) Study area; (**b**) Calendar year, vegetative phase, and reproductive phase of UK winter rapeseed defined in this study. (Adapted from^32^ with permission). NE stands for North East, NW stands for North West, YH stands for Yorkshire and the Humber, EM stands for East Midlands, WM stands for West Midlands, EE stands for East of England, SW stands for South West, Wal stands for Wales, NI stands for Northern Ireland, Sco stands for Scotland, SEL stands for South East and London. The same letters were used throughout this study.

This study focused on the arable lands (Fig. 1a) of the UK, between the latitudes 49° and 61° N and longitudes 9° W and 2° E. The large-scale shapefiles of the UK nations and England regions were downloaded from the UK Office for National Statistics^40,41^. Given this study simulates the stress and production loss since 1961, land cover map 1990 of the UK, which is the first national land use product, was obtained from Digimap and used other than recent ones^42^. The UK rapeseed fields packages between 2016 and 2024 growing seasons were downloaded from Digimap^43–51^. The rapeseed growing fields were produced by using Copernicus Sentinel 1 C-band Synthetic Aperture Radar and S2 (i.e. Sentinel-2) optical data (since 2016), with a minimum mappable area of 0.02 km^2^ (https://www.ceh.ac.uk/data/ceh-land-cover-plus-crops-2015). The fields layers were resampled to raster layers with a 100 m resolution. Documented rapeseed yields available for national and regional levels between 2016 and 2024 were obtained from Defra^52^.

The daily temperature data from the HadUK-Grid Gridded Climate Observations (1 km resolution, v1.2.0 (1836–2022)) were obtained in NetCDF format from the Centre for the Environmental Data Analysis (CEDA) archive^53,54^. Data covering the period 01/09/1960-31/07/2020 were used, corresponding to 1961–2020 rapeseed growing seasons.

The crop suitability index in classes (Fig. S3b) for current cropland in grid cell for the period 1961–1990 was obtained from the Global Agro-Ecological Zones v4 (GAEZ v4) model^55^. The used parameters for model generation of suitability class for rapeseed were rain-fed water supply and high input level. There are nine suitability classes (Table S1), and only land pixels inside the first six classes which permit at least 40% of maximum attainable yield of rapeseed and commercial rapeseed production were considered in this study. The crop (i.e., rapeseed) attainable yield (Y_attainable_) in global grids (Fig. S3d) was obtained from the website (https://gaez.fao.org/pages/data-access-download) published by FAO for Global Agro-Ecological Zones v4 (GAEZ v4)^55^, and the share of cultivated land (Fig. S3e) at the global scale was downloaded from the site (https://www.gaez.iiasa.ac.at/) provided by Global Agro-Ecological Zones v3 (GAEZ v3)^56^. Both the attainable yield and the share of cultivated land data were clipped to the UK land area. Observed rapeseed yield data are influenced not only by climate variability but also by changes in cultivars, management practices, fertilization and pest control. To isolate the long-term climatic signal and enable a spatially explicit assessment of heat stress-related production losses over 1961–2020, we therefore used attainable yield estimates from the GAEZ v4 framework. GAEZ v4 provides agro-ecological yield potentials based on standardized assumptions regarding crop management, soils, and climate, and has been widely used for national, regional, and global assessments of crop suitability and yield studies^57,58^. GAEZ v4 attainable yield data were used as a standardized agro-ecological reference baseline rather than as a predictor of observed yields, consistent with recent UK-scale land-use and crop productivity modelling studies^57,59^. While GAEZ v4 data are not intended to reproduce fine-scale or farm-level yield variability, they provide a consistent and internally comparable reference baseline for assessing relative spatial patterns and temporal variability in climate-induced production risk.

### Phenology of UK winter rapeseed

In the UK, it can take one to two weeks for rapeseed to emerge, and after flowering the crop completes pod development and ripening over a period of six to eight weeks^60^. The onset of reproductive development is generally associated with stem elongation, whereas the formation of florets and pods marks the subsequent progression of reproductive growth^61^. Flowering has been commonly used as a practical phenological marker to differentiate the pre-flowering growth period from the main yield-forming reproductive phase (from April to July)^62^. Winter rapeseed is normally sown in the UK from mid-August to mid-September; completes vegetative growth in November; develops the stem in February; produces first flowers in late March or April, the flowering period ends in May; pod and seed development is completed in late June and, finally, ripening and senescence occurs in July (Fig. 1b)^32,33,63,64^.

Therefore, the period from August 15th to July 31 st the following year was marked as the calendar year of UK rapeseed (Fig. 1b). The flowering period was set from the start of April to the end of May, capturing the peak flowering window across UK regions and accounting for south–north differences in phenological timing^3^. The defined rapeseed calendar year was aligned with the typical sowing period of UK winter rapeseed, which commonly occurs in August according to the AHDB’s Recommended Lists for cereals and oilseeds^25^. A similar calendar year configuration was used when investigating crop impacts of winter chilling or warming and crop monitoring of UK rapeseed^33,64^. For heat stress the present study focused mainly on flowering time, while for cold stress both vegetative and main yield-forming reproductive periods were additionally assessed^29,37^.

### Temperature thresholds and stress intensity indices (*f*_*HS*_ and *CDD*)

#### Temperature thresholds

To determine temperature stressed areas, it is a prerequisite to know this crop’s cardinal temperatures. In this regard, published peer-reviewed journal articles, reports from UK agronomic specialists (e.g., AHDB) were consulted and a value of 3℃ used as the base temperature (*T*_*base*_) for UK rapeseed crops, which is largely based on experimental outputs^31,36^. A temperature of 3℃ is also the minimum at which germination will occur^30^. Basu found that 15℃ permits the best germination of several varieties of UK rapeseed crops compared with both lower and upper temperatures^36^. This concurs with other peer reviewed sources and industrial reports that suggest optimum temperatures (*T*_*opt*_) of the growth of rapeseed crops in the UK, Italy and Hungary to be 15–25 °C^13,18,25,65,66^. Many studies have suggested that the *T*_*limit_h*_ of rapeseed crop is around 35 °C, which is the same as the *T*_*limit_h*_ of most UK crops^13,66^. Based on extensive previous work, the present study adopted *T*_*base*_/*T*_*cri_c*_, *T*_*cri_h*_, and *T*_*limit_h*_ temperatures of 3 °C, 25 °C and 35 °C for rapeseed grown across the globe (Table 1), excluding that the *T*_*cri_c*_ for December in each year which was set to be −3 °C (due to colder temperatures over this threshold benefiting rapeseed yields based on the study by^32^.

**Table 1:** Temperature (°C) thresholds used for the evaluation of temperature stress of rapeseed crop in the UK.

Crop	T_base_ (also as T_cri_c_, months excluding December)	T_cri_c_ (December)	T_opt_	T_cri_h_	T_limit_h_
Rapeseed	3 °C	−3 °C	15–25 °C	25 °C	35 °C
Refs.	^13,25,31,36,65,66^	^32^	^13,25,65,66^	Used as the critical high temperature of heat stress	^13,67^

#### Temperature stress indices (*f*_*HS*_ and *CDD*)

The heat stress index (*f*_*HS*_) was used based on previous frameworks demonstrating high temperature stress’ impacts on crop yields^1^. The basis of this methodology is that (1) rapeseed is sensitive to heat stress during the flowering phase and the whole of the reproductive stage; (2) damage to rapeseed plants initiates when maximum daytime temperatures (*T*_*max*_) exceed *T*_*crit_h*_; (3) and maximum damage is reached when *T*_*max*_ exceeds the *T*_*limit_h*_. The degree days method was used to compute cold stress expressed in cold degree days (*CDD*, unit: ℃ d)^5^, and with stress beginning when minimum daytime temperatures (*T*_*min*_) were below 3℃ (for the months other than December each year) or −3℃ (for December each year) (Table 1). Here, *CDD* is used as an agro-climatic indicator of reduced thermal availability for growth rather than a direct measure of frost injury, acknowledging that winter rapeseed can tolerate substantially lower temperatures following cold hardening.

To ensure the stress assessment covered arable land where rapeseed could be cultivated, only data in grid cells that were designated as arable in the Digimap land cover dataset were considered^42^. This was similar to the method which assessed the heat stress of crops only in areas with equal or over 20% attainable yields^1^. To do this, the areas of “arable and horticulture” in UK land cover map 1990 were identified and saved as “UK arable and horticulture land 1990” (Fig. S3a)^42^. Then the overlapping land pixels of UK arable and horticulture land 1990 and rapeseed suitability (resampled to the same spatial properties) were identified (Fig. S3c) and used as the study area of stress analysis, with the assumption of largest arable and suitable rapeseed cropping land. For simplification, the identified overlapping pixels were subsequently referred to as UK arable lands in this study and used for the extraction of temperature stress data. This method was used throughout this study, unless specified otherwise.

To calculate *f*_*HS*_ and *CDD*, their corresponding “daily” temperature stress intensity (*f*_*HS_d*_ and *CDD*_*d*_) as a function of daily temperatures (*T*_*day*_) were firstly assessed. Daily *T*_*max*_ was used to study heat stress as it has been observed that using *T*_*max*_ instead of daily mean temperature can obtain more accurate estimation, as the canopy temperatures often exceed air temperatures *T*_*max*_^68^. *T*_*min*_ was used for cold stress evaluation. The daily temperature stress intensity indices are shown in Eqs. (1 and 2):

1 $$f_{{HS\_d}}= \left\{ {\begin{array}{*{20}c} {~~~~~~~~~~~~~~~~~~~~~~~~~~0.0;~~when~T_{{day}} < T_{{cri\_h}} } \\ {~(T_{{day}} - T_{{cri\_h}} )/~(T_{{\lim it\_h}} - T_{{cri\_h}} );~~when~T_{{cri\_h}} ~ \le ~T_{{day}} ~ < ~T_{{\lim it\_h}} } \\ {~~~~~~~~~~~~~~~~~~~~~~~~~~1.0~;~~~when~T_{{day}} \ge T_{{cri\_h}} } \\ \end{array} } \right.$$

2 $$CDD_{d}= \left\{ {~~~\begin{array}{*{20}c} {~~~~~~~~~~~~~~~~~~~~0;~~~T_{{day}}> ~T_{{cri\_c}} ~~} \\ {T_{{day}} - T_{{cri\_c}} ;~~~T_{{day}} ~ \le ~T_{{cri\_c}} ~~} \\ \end{array} } \right.$$

where *f*_*HS_d*_ is the daily values heat stress index, *T*_*day*_ is the input temperature which is *T*_*max*_ *or T*_*min*_, *T*_*cri_h*_ is the critical high temperature threshold, *T*_*limit_h*_ is the limit/ceiling high temperature threshold, *CDD*_*d*_ is the daily values of cold degree days, *T*_*cri_c*_ is the critical low temperature threshold, *T*_*min*_ is the minimum daytime temperature.

The daily heat stress index (*f*_*HS_d*_) was then summed throughout the heat-sensitive period (*HSP*), which was identified as the flowering months in April and May. The obtained values were divided by 61 days (number of days during the *HSP*), to calculate the averaged stress intensity index *f*_*HS*_ (as shown in Eq. 3) for each year. This step allows the calculated values of heat stress to represent both the intensity and number of stressful days rapeseed plants would have experienced. Since temperature extremes during pod and seed development (for rapeseed in the UK the period includes June and July as shown in Fig. 1) can also have negative impacts (such as preventing photosynthesis, decreasing oil accumulation, etc.) in rapeseed crops differentially between genotypes^10^, *f*_*HS*_ in June and July in each year was also assessed.

3 $$f_{{HS}}= \mathop \sum \limits_{{i = 1}}^{{HSP}} f_{{HS\_d}} /HSP$$

where *f*_*HS*_ is the heat stress index, *HSP* is the number of days in heat-sensitive period, *f*_*HS_d*_ is the daily heat stress index.

Similar to the case of obtaining *f*_*HS*_, the *CDD*_*d*_ were summed to compute the *CDD* (Eq. 4) of each study period, with the exception that *T*_*cri_c*_ of December of each year was set to −3 °C^32^. Then pixel-level *f*_*HS*_ and *CDD* for each studied crop stage (flowering, vegetative and reproductive phase (from April to July) were computed on an annual basis from 1961 to 2020 rapeseed calendar years. The spatiotemporal patterns of *CDD*, *f*_*HS*_, and percentages of days experiencing the stresses were analysed at the annual pixel and decade levels for the studied period (i.e. 1961–2020). The annual scale analysis focused on studying pixel-level trends and variabilities of the stress, using stress values normalized by the maximum annual mean stress across all land pixels over 1961–2020. The decadal level analysis examined the trends of maximum stress of each decade. Additionally, the annual values were used to calculate the minimum, maximum, mean, and standard deviation across the decades over 1961–2020 at the UK scale. To facilitate the comparison of stress, the heat stress during flowering stage was also classified into five stress levels including very low (*f*_*HS*_ = 0), low (0 < *f*_*HS*_ < 0.05), medium (0.05 ≤ *f*_*HS*_ < 0.15), high (0.15 ≤ *f*_*HS*_ < 0.30), and very high (*f*_*HS*_ ≥ 0.30) stress^1^. A pixel-wise linear regression model was applied to annual mean values (stress intensity and percentages of days experiencing stress) of *f*_*HS*_ and *CDD* to investigate the trends of heat stress and cold degree days across calendar years.

4 $$CDD = \mathop \sum \limits_{1}^{n} CDD_{d}$$

where *CDD* is the accumulated value of daily values of cold degree days over stressful days, *n* is the number stressful days for each type of studied periods, *CDD*_*d*_ is the value of daily cold degree days.

### Normalized rapeseed production loss index (*f*_*RPL*_)

In addition, this study followed the study by Teixeira and coworkers^1^ and developed a rapeseed production loss index (*f*_*RPL*_) for assessing crop loss as a result of heat stress (Eq. 5), built on the available data of crop attainable yield (*Y*_*attainable*_) (Fig. S3d) and the share of cultivated land (based on global land cover data for the year 2000 and several related dataset) for UK grids (Fig. S3e) in GAEZ v4 and GAEZ v3, respectively^55,56^. The parameters used to retrieve attainable yield were: Average attainable yield of current cropland, for the period 1961–1990, rapeseed under rainfed conditions, high input level, with CO_2_ fertilization, climate data source CRUTS32 based on historical data for the time periods 1981–2010. The parameters used to obtain the share of cultivated land was cells with a fraction of cultivated land at least 5%. Both the attainable yield and the share of cultivated land in the two platforms are in 5 arc-minute resolution (approximately 6.7 km at 44°), which was processed into 1 km resolution to be comparable with the resolution of stress index layers. The crop attainable production (as dry matter in kg) of each gird cell was calculated by multiplying the attainable yield (kg km^− 2^), crop area/grid cell size (km^2^) and share of cultivated area (%).

The annual production loss index (*f*_*RPL_ag*_) of each grid cell was assessed as the product of attainable production and *f*_*HS*_ of the same year (Eq. 5), and the grid level maximum *f*_*RPL_ag*_ (*f*_*RPL_ag_max*_) over the period 1961–2020 was identified. To enable the comparison of magnitude and annual variability of heat impact to rapeseed production, the normalized production loss index (*f*_*RPL_ng*_) of each pixel over the sixty-year study period (e.g., 1961–2020) was obtained by normalizing each *f*_*RPL_ag*_ with *f*_*RPL_ag_max*_ (Eq. 6)^1^. The annual mean *f*_*RPL_n*_ (*f*_*RPL_nm*_) of all land pixels for the UK and its eleven regions (South East and London were regarded as one region as the case of regional rapeseed yield dataset were calculated), and a simple linear model was used to fit their trends^69^. A single factor Analysis of Variance (ANOVA) analysis followed by Tukey’s honesty significant difference (HSD) test were applied to the *f*_*RPL_nm*_ of the UK and its regions, with between-decade analysis (i.e. using data points of all pixels for each decade) and between-region analysis (over the period 1961–2020 or crossing decades) were conducted.

5 $$f_{{RPL\_ag}} = Y_{{attainable}} ~ \times ~Cultivation~area~ \times ~\% ~of~cultivation~land~ \times ~f_{{HS}}$$

6 $$f_{{RPL\_n}} = \mathop \sum \limits_{{i = 1~}}^{n} f_{{RPL\_ag}} /f_{{RPL\_ag\_\max }}$$

where *f*_*RPL_ag*_ is the production loss index of each grid cell of each year, *Y*_*attainable*_ is the attainable yield of rapeseed crop, *f*_*HS*_ is the annual heat stress index of each grid cell, *f*_*RPL_n*_ is the normalized production loss index of rapeseed of each grid cell in each year, *n* is the number of grid cells, *f*_*RPL_ag_max*_ is the maximum value of *f*_*RPL_ag*_ of all grid cell during 1961 and 2020.

### Regression relationships between heat stress and rapeseed yields during the period 2016–2024

To validate the modelled negative impacts of heat stress on rapeseed productivity, using available rapeseed fields and yields data of the calendar years from 2016 to 2024, the regression relationships between estimated mean heat stress intensity and rapeseed yields at the UK national and regional levels were examined. Then a simple linear regression model was applied.

### Statistical analysis

R version 4.2.3 was used for data processing, analysis and visualization^70^. The violin plot and ggplot2 boxplot function were used to visualize decadal *f*_*RPL_n*_, and boxplot was used for the visualization of regional *f*_*RPL_n*_^71^. R^2^ (i.e. the coefficient of determination) was used to assess the strength of trends for the intensities and percentages of days experiencing heat stress and cold stress from 1961 to 2020 at the pixel level. The annual mean values of *f*_*HS*_, *CDD*, *f*_*RPL*_ at the UK scale were used to fit linear models to study their temporal trends during the studied period. The R^2^ and p values were used to evaluate the goodness of established regression model between rapeseed yields and *f*_*HS*_ over 2016–2024. The calculation for R^2^ is shown in Eq. (7). The values of R^2^ were categorised into four classes including very weak (0 ≤ R^2^ < 0.25), weak (0.25 ≤ R^2^ < 0.5), strong (0.5 ≤ R^2^ < 0.75), very strong (0.75 ≤ R^2^ ≤ 1) for assessing trends in this study.

7 $$R^{2} = 1 - \frac{{\Sigma _{{i = 1}}^{N} \left( {y_{i} - f_{i} } \right)^{2} }}{{\Sigma _{{i = 1}}^{N} \left( {y_{i} - \bar{y}} \right)^{2} }}$$

where y_i_ is the ith observed value, $\:\stackrel{-}{\mathrm{y}}\:$ is the mean of observed values, f_i_ is the fitted value for y_i_ and N is the total number of observed data. In the best case with a R^2^ value of 1 indicates the modelled values can perfectly match the observed values, the higher the R^2^, the greater explanatory power of the model.

## Results and Discussion

### Cold degree days (*CDD*) and beneficial cold in December

**Fig. 2:**
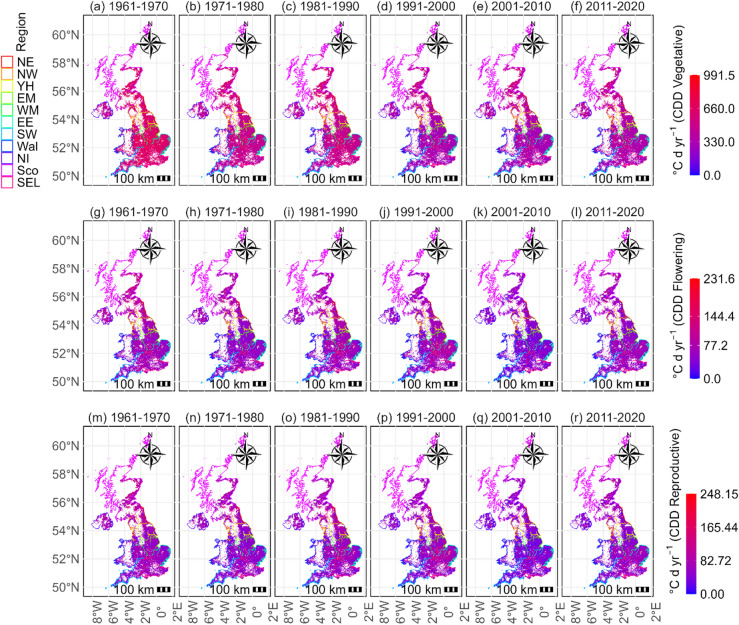
Maximum *CDD* of UK arable lands for rapeseed cropping during (**a**–**f**) vegetative, (**g**–**l**) flowering stage and (**m**–**r**) reproductive stages of each decade during 1961–2020.

Because cold temperatures could impact the distribution and productivity of crops, arable areas affected by cold stress for UK rapeseed cultivation were analysed. Figure 2 shows the trends of *CDD* values on potential UK rapeseed cropping land for the vegetative, flowering and reproductive periods during 1961–2020. For the flowering period, the results indicate that cold stress has been generally decreasing with temporal variations throughout the studied period (Fig. 2g–l). The decrease in cold stress can also be seen from the decreasing percentages of days experiencing cold stress for the corresponding periods (Fig. S4g–l). Temporally, more and more UK lands had smaller *CDD* values, and the magnitude of cold stress in the south of the UK was smaller than in the north (Fig. 2g–l), with an increasing trend over time (Fig.S5e,f). The decrease in cold stress, which was more observable at the pixel scale where negative slope values for *CDD* were observed (Fig. S5e,f), also showed noticeable interannual variations (R^2^ values being lower than 0.50 as shown in Fig. S5g). Although negative slopes indicate a general decline in cold stress over time, the low R² values highlight strong interannual variability, suggesting that these trends represent gradual long-term tendencies rather than dominant drivers of year-to-year variability. Figure 2g-l also illustrated that cold stress in England, where most UK rapeseed is cultivated, was generally lower than stresses in the north^72^. The percentages of days under cold stress for each development phase were also slowly decreasing for most areas at the pixel level (Fig. S7a–c).

Table 2 shows the summary of stress values of all land pixels at the UK national scale. The *CDD* values of the flowering period demonstrated a declining trend (Table 2). The ranges of percentages of days experiencing cold stress also exerted decreasing mean values (Table 2). In addition, cold stress values of UK arable soils for rapeseed cropping during its vegetative and reproductive phases on a decadal basis from 1961 to 1970 to 2011–2020 were studied. Results showed that overall cold stress declined throughout the studied periods, with values of *CDD* during vegetative periods were larger than those of reproductive phases (Table S2). For the same periods the percentages of cold stressed days had decreasing mean values (Table S2). Regarding the reproductive periods, the *CDD* were also decreasing over the studied period (Table S2). These decreasing trends were the same as the flowering period (Fig. 2; Table 2). Compared to the significant decrease of most lands across the UK in vegetative periods (Fig. S5d), the decrease was not significant during the flowering and reproductive periods, especially for southern regions (Fig. S5h,i).

**Table 2:** Cold stress and heat stress values of UK arable lands for rapeseed cropping.

Periods	1961–1970	1971–1980	1981–1990	1991–2000	2001–2010	2011–2020
f_HS_ (min – max); flowering	0–0.009	0–0.013	0–0.022	0–0.028	0–0.016	0–0.023
f_HS_ (mean ± sd); flowering	0.001 ± 0.001	0.002 ± 0.002	0.003 ± 0.003	0.002 ± 0.003	0.003 ± 0.003	0.004 ± 0.003
Percentage (%) of days experiencing heat stress (min – max); flowering	0–4.73	0–6.56	0–11.48	0 − 16.39	0–9.18	0–13.11
Percentage (%) of days experiencing heat stress (mean ± sd); flowering	1.13 ± 0.92	1.76 ± 1.53	2.7 ± 2.31	3.1 ± 2.86	2.67 ± 1.76	4.21 ± 2.51
CDD (min – max); flowering	6.92–211.60	9.18–210.05	6.46–184.36	4.75–181.16	4.22–167.27	8.45–200.76
CDD (mean ± sd); flowering	19.45 ± 4.71	20.62 ± 5.59	21.79 ± 4.88	20.16 ± 4.45	17.43 ± 4.85	18.13 ± 4.51
Percentage (%) of days experiencing cold stress (min – max); flowering	5.96–51.62	6.59–56.43	5.74–47.77	3.65–49.82	3.28–43.39	4.92–51.29
Percentage (%) of days experiencing cold stress (mean ± sd); flowering	67.24 ± 20.18	57.36 ± 19.48	64.65 ± 21.03	62.72 ± 20.41	42.31 ± 16.09	57.43 ± 16.56
f_HS_ (min – max); June and July	0–0.073	0–0.259	0–0.183	0–0.164	0–0.231	0–0.228
f_HS_ (mean ± sd); June and July	0.021 ± 0.012	0.11 ± 0.062	0.057 ± 0.031	0.055 ± 0.031	0.094 ± 0.051	0.079 ± 0.046
Percentage (%) of days experiencing heat stress (min – max); June and July	0–33.53	0–62.30	0–54.10	0–45.90	0–57.38	0–59.26
Percentage (%) of days experiencing heat stress (mean ± sd); June and July	11.24 ± 6.30	31.47 ± 13.26	24.4 ± 9.91	22.33 ± 10.01	32.73 ± 12.73	31.54 ± 13.74

*min* minimum, *max* maximum, *sd* standard deviation. The unit of CDD: ℃ d yr^− 1^.

**Fig. 3:**
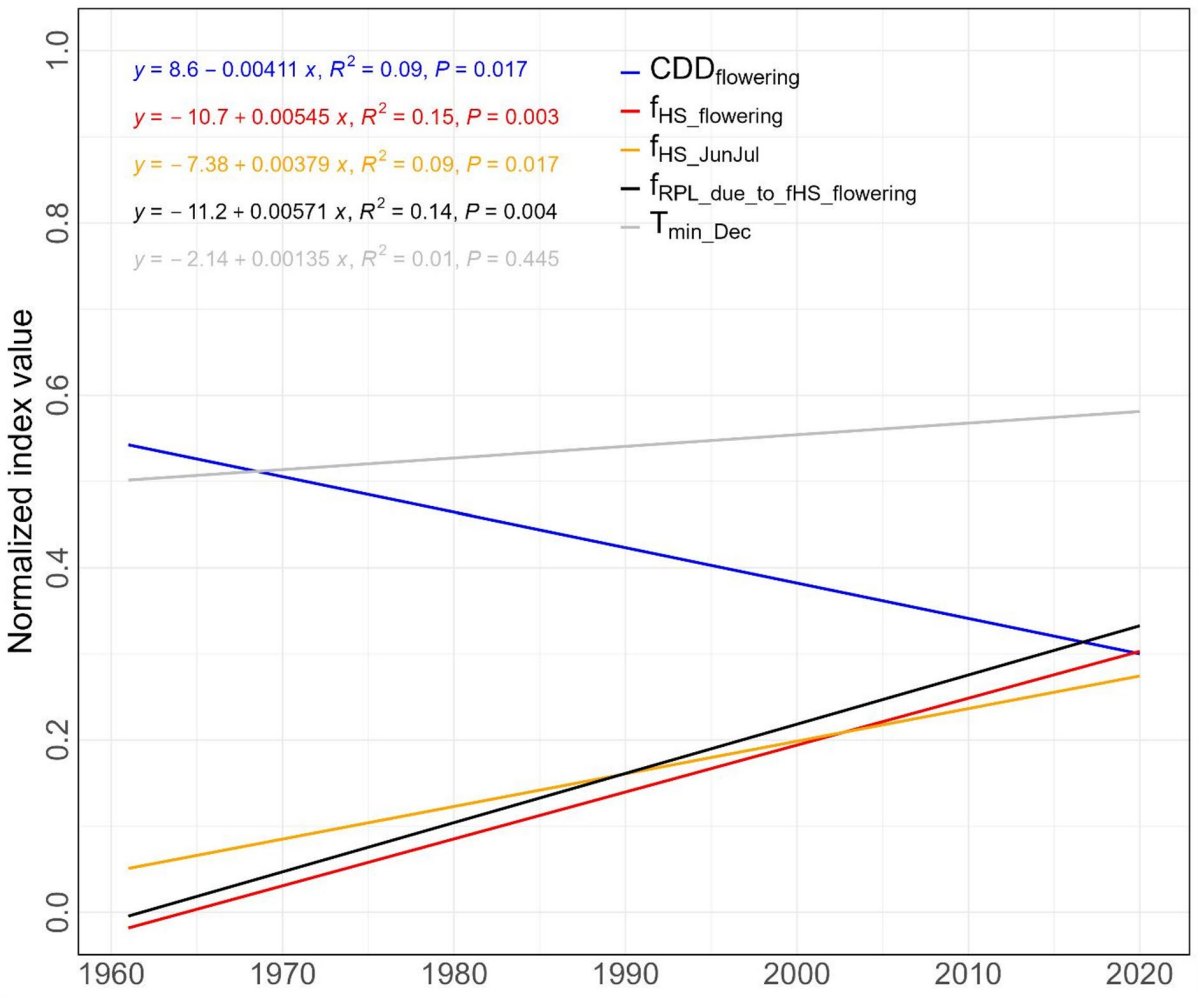
Fitted trajectories of *f*_*HS*_ during flowering and during June and July, *CDD* values during flowering, *f*_*RPL*_ due to *f*_*HS*_ during flowering, and mean *T*_*min*_ of Dec over 1961–2020. Data were shown in normalized index.

Given colder temperatures yet still warmer than − 3 °C during December could benefit UK rapeseed yield, the fitted trajectory of *T*_*min*_ of December on potential UK rapeseed cropping land from 1961 to 2020 was analysed^32^. There was a tendency of slow increase in *T*_*min*_ (Fig. 3) with anomalies between *T*_*min*_ of 1980–2010 (Fig. S6) and a fluctuating trend at the UK level.

### Heat stress (*f*_*HS*_)

**Fig. 4:**
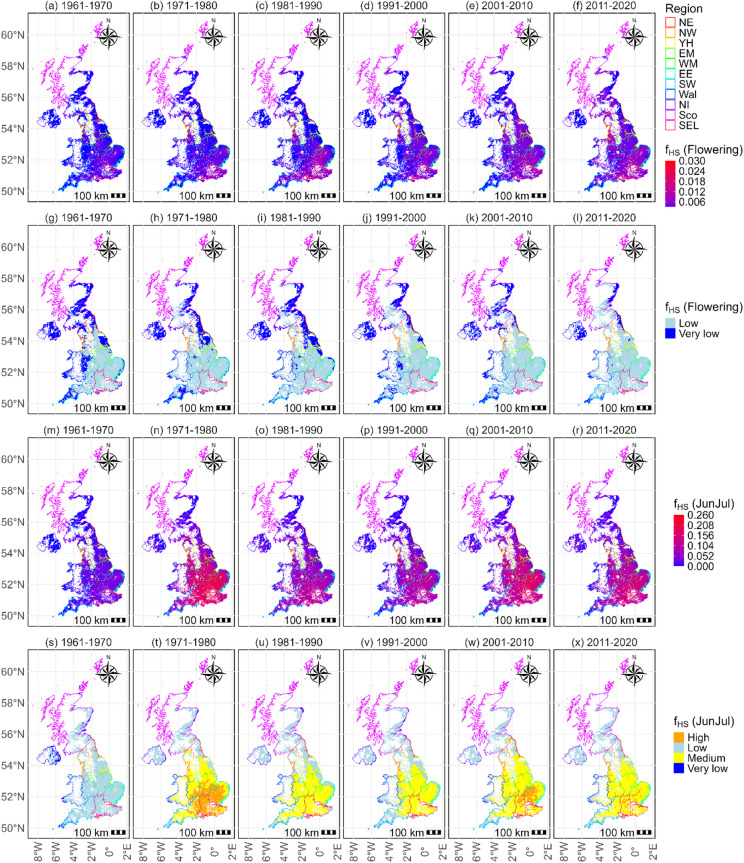
Maximum *f*_*HS*_ of UK arable lands for rapeseed cropping during flowering stage and June and July. (**a**–**f**) *f*_*HS*_, (**g**–**l**) *f*_*HS*_ in class (**m**–**r**) *f*_*HS*_, (**s**–**x**) *f*_*HS*_ in class. JunJul indicates the period June and July. Heat stress levels are very low (*f*_*HS*_ = 0), low (0 < *f*_*HS*_ < 0.05), medium (0.05 ≤ *f*_*HS*_ < 0.15), high (0.15 ≤ *f*_*HS*_ < 0.30), and very high (*f*_*HS*_ ≥ 0.30) stress intensity.

Figure 4 shows the trends of *f*_*HS*_ values of potential UK rapeseed cropping land for the flowering, and June and July for each decade over 1961–2020. There was a general increasing trend of intensities (Fig. 4a–f and m–r) for both developmental periods, with the southern UK having higher levels of stress. For the flowering phase, the areas without experiencing very low level (*f*_*HS*_ = 0) of heat stress were decreasing and areas impacted by low level of stress showed an increasing trend (Fig. 4g–l). The mean values of *f*_*HS*_ in each decade were greater over time (Table 2). Spatially the southeastern of the UK had higher *f*_*HS*_ values compared with the northern and western parts (Fig. 4), with the mean *f*_*HS*_ intensities for the flowering period of each decade being 0.001 ± 0.001, 0.002 ± 0.002, 0.003 ± 0.003, 0.002 ± 0.003, 0.003 ± 0.003, 0.004 ± 0.003. The slowly increasing heat stress was also observable in Fig. S8a–f, which demonstrated pixel-wise slow increase of percentages of days experiencing heat stress from 1961 to 1970 to 2011–2020. The maximum value of the percent of days were increasing, and their mean values followed the same trend (Table 2). The stress intensities and areas impacted for the period June and July (Fig. 4m–x; Table 2) were greater compared to the flowering period (Fig. 4a–l). Most areas appeared to experience medium level of stress for this period and the areas experiencing high level stress were increasing (Fig. 4s–x). Fig. S9a–d show a generally increasing trend in annual *f*_*HS*_ during the flowering period from 1961 to 2020, indicating a gradual intensification of heat stress, although the low R² values (≤ 0.20) suggest substantial uncertainty in these trends. Spatially, smaller or near-zero increases occurred in Scotland, Northern Ireland, the South West and North East, while stronger and in some areas significant increases were observed mainly in the West Midlands, East Midlands, North West, and Yorkshire and the Humber, particularly across the East of England and South East.

The annual patterns of pixel-wise *f*_*HS*_ for June and July over 1961–2020 and the associated R^2^ and p values of the increase are shown in Fig. S9e–h. The pixel-wise trends of heat stress of this phase depict faster increasing trends of *f*_*HS*_ over a larger area than during flowering, and exhibited larger variations (Fig. S9). Similar to the conditions in flowering, most land experienced increasing stress, with significant increases observed for most regions excluding South West, Wales, Scotland, and Northern Ireland. The spatiotemporal maps of *f*_*HS*_ (Fig. 4m–r) and percentages (Fig.S8g-l) of June and July also demonstrate increasingly severe stress impacts across wider areas. The maximum values of *f*_*HS*_ and percentages of days experiencing heat stress of each decade between 1961 and 2020 increased generally from 4.45 to 13.93 and from 33.53% to 59.26%, respectively, with their mean values were also increasing (Table 2). The percentages of days experiencing heat stress for the flowering period and June and July (Fig. S7d,e) also showed a slowly increasing trend for most areas. Overall, these results indicate a slow but spatially coherent increase in heat stress across most UK arable lands, with stronger trends in south-eastern regions and substantial interannual variability.

### Normalized rapeseed production loss index (*f*_*RPL_n*_)

**Fig. 5:**
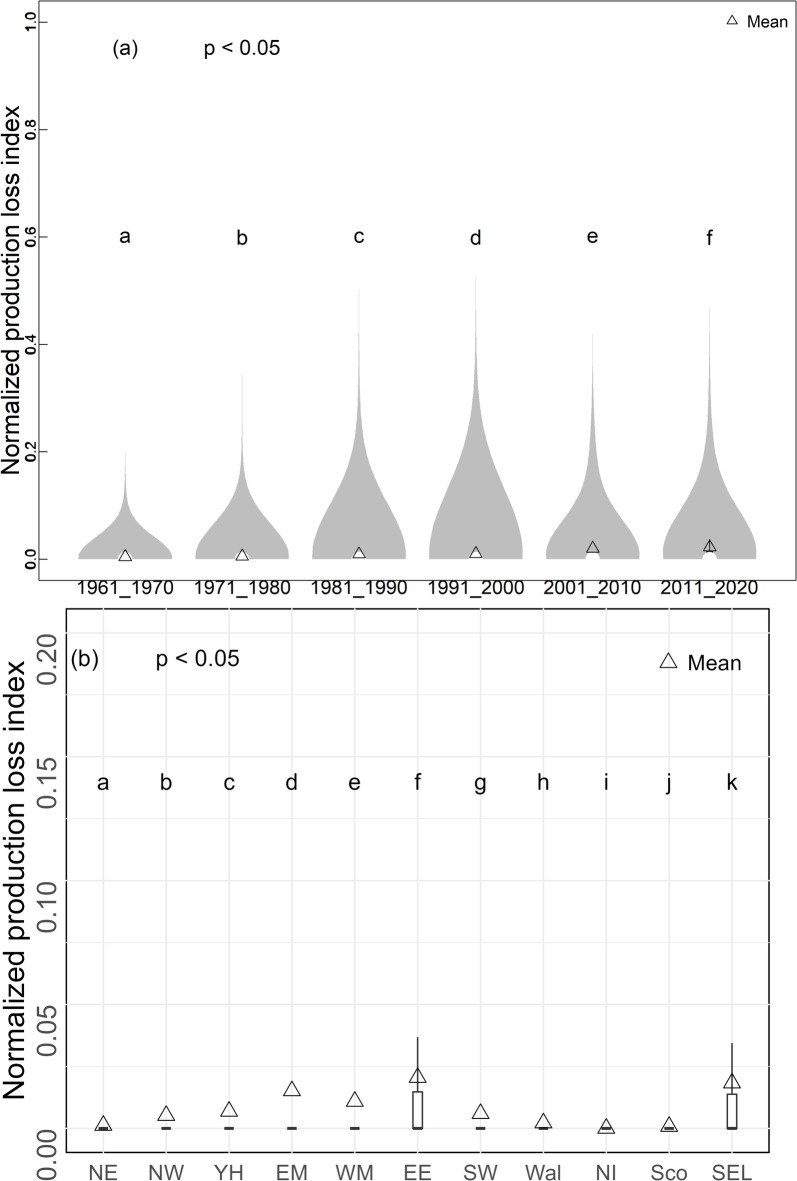
(**a**) The *f*_*RPL_n*_ due to heat stress of all grid cells in flowering period for the UK during six decades between 1961 and 2020 (Box-boundaries were invisible due to the 25th and 75th percentiles were zero), and the violin plot depicts the distributions of normalized pixel level *f*_*RPL_n*_; (**b**) *f*_*RPL_n*_ of all grid cells for each UK region during the flowering period between 1961 and 2020. Box-boundaries were the 25th and 75th percentiles, and the triangles show mean values. The lowercase letters indicate statistically significant differences among regions tested by ANOVA with Tukey’s HSD test, with no significant differences between the regions having the same letter.

The *f*_*RPL_ng*_ due to heat stress during flowering for all grid cells of arable lands in each ten-year period between 1961 and 2020 is shown in Fig. 5a. At the UK level, there was an increasing mean value (Fig. 5a), although hardly noticeable, of *f*_*RPL_ng*_ over the studied periods, which is consistent with the increases in intensity (Fig. 4a–l and Fig. S9a) and frequency (Fig. S8a–f) of heat events. The 25th and 75th percentiles of *f*_*RPL_ng*_ for each 10-year period were all zero, explaining that they were not visible in Fig. 5a. The year 2010 had the highest normalized production loss index (data not shown here) which means the impact of heat stress on rapeseed cropping was largest in this year. Additionally, there was an increase (*p* < 0.001) of *f*_*RPL_nm*_ in rapeseed resulting from heat stress (Fig. S10). The ANOVA analysis indicated significant difference (*p* < 0.05) in *f*_*RPL_nm*_ of rapeseed between decades (Fig. 5a) and UK regions (Fig. 5b). Notably, the largest *f*_*RPL_n*_ (Fig. 5b) was observed in the five regions (including East of England, East Midlands, Yorkshire and the Humber, South East and London, and West Midlands shown in Fig. S1b), which were also the regions with the largest rapeseed cropping areas in the recent decade.

**Fig. 6:**
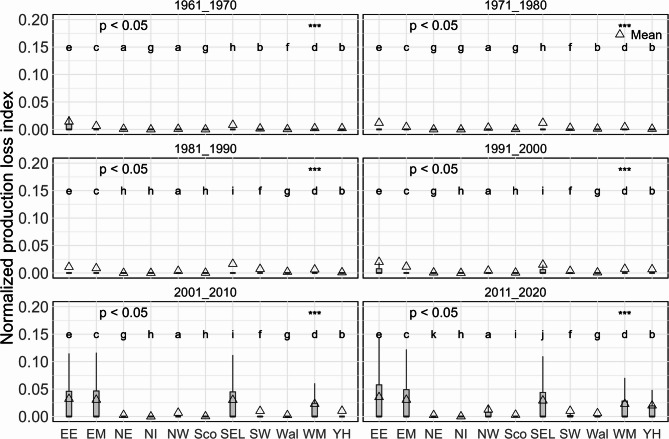
Normalized rapeseed production loss index (*f*_*RPL_n*_) due to heat stress of all grid cells in flowering period for UK regions of each decade between 1961 and 2020. Box-boundaries are the 25th and 75th percentiles, and the triangles show mean values. The asterisk “***” denotes significance levels corresponding to p-value of 0.001.

While Fig. 5 showed that there was a difference in *f*_*RPL_n*_ across regions over 1961–2020, more detailed information about whether there is a difference in *f*_*RPL_n*_ for each region for each decade was lacking. Given this, analysis was conducted to fill this gap. Figure 6 shows the *f*_*RPL_n*_ by regions by decades, revealing significant differences of the production loss between regions across decades and low levels of *f*_*RPL_n*_ before 1990s. However, the boxplots show that there were visible *f*_*RPL_n*_ since 1991–2000, particularly for two regions (East of England, and South East and London). In addition, East Midlands, West Midlands and Yorkshire and the Humber had a noticeable increase in *f*_*RPL_n*_ from 1991 to 2000. These observations demonstrate that the primary rapeseed production regions are experiencing risk of heat stress. Additionally, at the annual scale over 1961–2020, *f*_*RPL*_ (i.e. the potential rapeseed production loss index) and *f*_*HS*_ during flowering showed the largest increasing trend (*p* < 0.05) (Fig. 3). The increase in heat stress for flowering was faster than not only the later reproductive period (June and July), but the decrease in cold stress of the flowering period (Fig. 3).

### Regression relationships between heat stress and documented rapeseed yields during the period 2016–2024

**Fig. 7:**
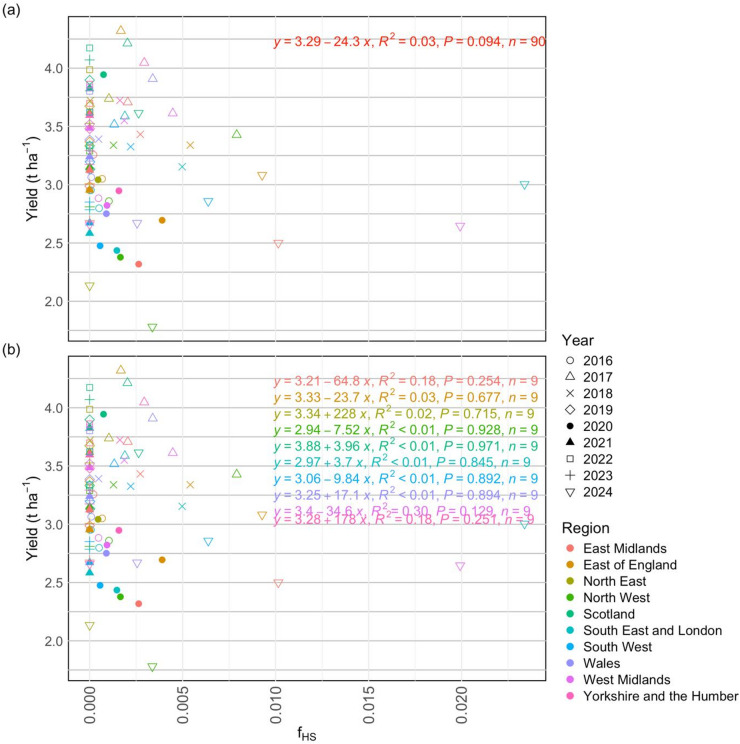
Linear regression relationships between rapeseed yields and mean values of heat stress during flowering period of 2016–2024 at the UK (**a**) national and (**b**) regional levels.

Figure 7 shows the regression relationships between rapeseed yields and mean *f*_*HS*_ intensity during flowering of 2016–2024 at national and regional levels. At the national scale (Fig. 7a), yields tend to decrease with increasing heat stress intensity, although the relationship being very weak (R^2^ = 0.03) and not statistically significant (*p* > 0.05). For regions (Fig. 7b), relatively stronger but still weak relationships were observed (R^2^ ≤ 0.30), with none reaching statistical significance (*p* > 0.05). The direction of these associations varied across regions, with weak positive or negative relationships observed along a latitudinal gradient, potentially reflecting historical temperature constraints in different rapeseed-growing areas. Among the primary rapeseed growing regions (Fig. S1b), which were also modelled to experience greater heat stress and higher production risk (Fig. 6), the East of England, East Midlands and West Midlands exhibited negative relationships (*p* > 0.05). Nevertheless, the remaining regions showed very weak positive relationships between heat stress and rapeseed yields (Fig. 7b).

**Fig. 8:**
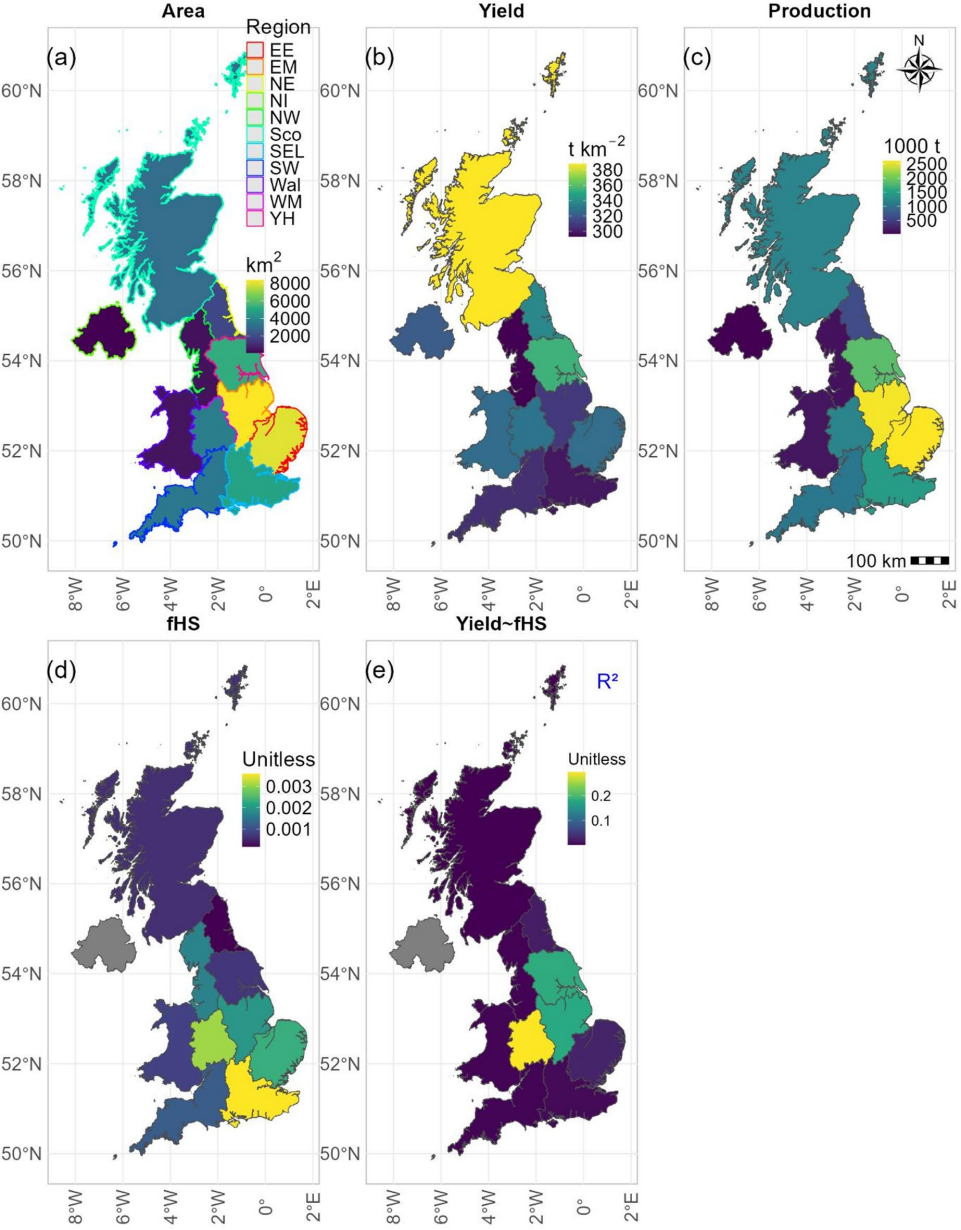
Statistics for UK rapeseed during the period 2016–2024. (**a**) Total cultivation areas, (**b**) Mean of annual yields (1 t km^− 2^ = 0.01 t ha^− 1^), (**c**) Total productions, (**d**) *f*_*HS*_, (**e**) R^2^ (i.e., the coefficient of determination) between rapeseed yield and *f*_*HS*_ for UK regions.

Additionally, while for 2016–2024 the regions having the largest total cultivated areas and total production (Fig. 8a,c) of rapeseed and modelled to experience greater risk of heat stress (Fig. 8d), the regression relationships between documented rapeseed yields and heat stress intensities (*f*_*HS*_) were relatively weaker compared to regions particularly West Midlands with smaller rapeseed fields (Fig. 8e). This implies a smaller sensitivity of rapeseed yields to increasing temperatures in primary rapeseed growing regions.

### Discussion

#### Increasing heat stress and decreasing cold stress on UK land for potential rapeseed cultivation

Temperature stress analyses demonstrate increased intensity and frequency of heat stress during both flowering and the whole reproductive period for UK arable lands over 1961–2020, implying an expanding area impacted by a warming climate. Southeastern regions of the UK experienced higher levels of heat stress than other regions (Fig. 4a–f). The overall small values of the slopes of heat stress during flowering indicated a mild increase of heat stress (Fig. S9a). Spatially, the increasing heat stress was less evident in the north (mainly Scotland and Northern Ireland) and South-western England (Fig.S9a). Not only the stress values were higher in south-eastern England (Fig. 4a–f), but these regions were also showing higher increasing trends (Fig. S9a), suggesting a concerning trajectory for rapeseed at these locations. This pattern aligns with previous studies showing that parts of England are increasingly exposed to temperatures exceeding 35 °C under climate change^73^. While warmer April months with temperatures below 15 °C may benefit UK rapeseed yielding, undesirable warming is detrimental^9,32,74,75^. The increasing exposure to temperature above 25 °C during flowering implies production loss of rapeseed in the UK, which was observed through the rapeseed production loss index (Figs. 3 and 5)^13,25,65,66^.

Additionally, Fig. 4m–x shows higher heat stress on UK arable lands during June and July compared to the flowering stage, with heat stress values (Fig. S8g–l) consistently greater in these months. However, the increasing trends in heat stress during June and July were lower than those during flowering (Fig. 3), indicating that while pod/seed development and ripening will face rising heat stress, it will not increase as rapidly as during the flowering stage. The number of land pixels not experiencing heat stress during the flowering period has generally decreased over the study period (Fig. 4g–l; Fig. S8a,b), reflecting a slow decline in suitable areas for rapeseed cropping in the UK. This trend aligns with predictions of decreasing suitable cultivation areas for rapeseed in the EU, including the UK, due to climate change^76^, although rapeseed’s early flowering time offers an inherent advantage in coping with heat stress^38^.

The findings of cold stress assessments revealed a decreasing pattern for all developmental periods (Fig. 2, Figs.S4 and S7a–c), though latitudinally the northern regions had higher cold stress values. Consistent with the present study, a model study suggested that cold stress has been reducing for rapeseed crop and it will decrease in most regions over the period 2040–2059 under the high-emission scenario^18^. In addition, chilling stress appeared to be reducing for rice cropping for most regions in China during 1960–2009 and there was also a decline of cold risk for maize from 1951 to 2015 in northeastern China^39^.

This study differs from a multi-crop study by analysing both heat and cold stress mainly over the whole of the flowering period, providing a deeper understanding regarding the impacts on rapeseed productivity of both the high temperature and low temperature extremes^1^. However, the *CDD* analysis may still underestimate the cold risk, as harmful chilling may start at temperatures above 3 °C. For example, cold stress (at 7 °C) during flowering led to slightly yield loss in winter rapeseed^6^. The *T*_*cri_c*_ of December was set as −3 °C due to the beneficial effect of early winter chilling for UK’s rapeseed yields^32^. The present study found more warm Decembers in recent years (Fig. S6), implying reduced cold periods. Moreover, unlike previous studies^5,18^, we demonstrated a faster increase in heat stress than a decrease pattern in cold stress (Fig. 3). This suggests the increasing heat stress during flowering period is of greater concern.

#### Increasing potential rapeseed production loss due to heat stress

The rapeseed production loss index enables the investigation of the magnitudes and annual variability of negative impact of heat stress on rapeseed cropping. Results demonstrated an increasing risk, which were significantly different between decades and UK regions (Figs. 5 and 6). The loss index analysis by region and by decade revealed that the two regions (East of England, and South East and London) had the largest loss for all studied decades (Fig. 5b). Apart from the two regions, a significant increase in the loss index of East Midlands, West Midlands and Yorkshire and the Humber since 2000 s was observed (Fig. 6), showing the increasing risk with implications extending to broader rapeseed cropping areas.

Furthermore, rising temperatures beyond 2020 are expected to further reduce UK rapeseed production, consistent with projections of a 6.1% productivity decline under the IPCC RCP8.5 scenario for 2050–2099 due to shortened crop growth cycles in the EU^77^. These findings highlight the need for further evaluation of climate stress impacts on UK rapeseed yields using stress data from actual rapeseed fields and observed yield statistics, with particular focus on major production regions identified as facing higher risk (Fig. 6)^38^.

#### Present UK rapeseed yields demonstrated resilience to heat stress whilst warming under climate change increases yield vulnerability

While the above discussion highlights the increasing heat stress and its negative impacts on potential rapeseed production, analysis showed that the regression relationships (Fig. 7a,b) between *f*_*HS*_ and reported rapeseed yields were very weak (*p* > 0.05) at both the national and regional levels. Although both being very weak, the relatively stronger relationships at the regional level indicate regions are showing different levels of sensitivity to temperature, which negate each other when assessed at the national scale. Notably, a negative relationship (which were observed in East of England, East Midlands and North East shown in Fig. 7b) between *f*_*HS*_ and rapeseed yields was observed, agreeing with previous studies^74,78^. On the other hand, greater *f*_*HS*_ were found to weakly and positively contributed to higher yields of several UK regions including those (Yorkshire and the Humber, South East and London, and West Midlands) major rapeseed production regions (Fig. 7b). This weak positive relationship is probably due to these UK regions being temperature limited^73,79^, where a warmer spring provides more beneficial growing environments for rapeseed plants during the flowering period over 2016–2024. However, it should be noted that the positive relationships captured here do not contrast the general conclusion that heat stress during critical period, such as the flowering phase, reduces crop yields and production once a threshold temperature is exceeded^74,78^. Despite the observed increasing trends in heat stress, the low coefficients of determination (R² ≤ 0.30) indicate that heat stress currently explains only a small proportion of the variability in documented UK rapeseed yields during 2016–2024. This suggests that water availability and other non-climatic factors—including cultivar selection, agronomic management, fertilization, and pest control—remain the dominant drivers of yield variability over this period. Consequently, while warming increases the vulnerability of rapeseed production, present-day yields in the UK still exhibit a degree of resilience to heat stress.

The relatively greater sensitivities (Fig. 8e) to increasing temperatures of smaller rapeseed land use and lower heat stress intensities regions than primary production regions in South-eastern UK indicates a spatially heterogeneous impact of warming on crop productivity^80^. These analyses demonstrated the complex and region-specific impacts of warming on rapeseed growth, development and productivity. Moreover, UK rapeseed yields currently exhibit short-term buffering capacity against warming, consistent with recent modelling studies for other UK crops such as wheat before the 2050s^79,81^. However, the warming climate is expected to reduce the resilience for rapeseed and other UK crops, underscoring the importance of ongoing research on breeding scheme (to identify cultivars with greater heat resistance), adaptive/mitigation strategy (film antitranspirant, etc.), and other agricultural management practices such as sowing date shifts^33,75,81–84^. For instance, a recent study focusing on UK rapeseed has found that twenty-eight genotypes were heat stress resistant and five genotypes had high yielding ability even under heat stress^75^. Beyond temperature stress alone, other climatic factors may further modulate crop responses to warming. Previous studies have shown that rainfall alone often exhibits weak or inconsistent relation with rapeseed yield, reflecting the importance of rainfall timing, soil water storage, and crop evapotranspiration demand rather than total rainfall amounts^85,86^. Soil water availability also plays a critical role in regulating crop thermal stress by influencing transpiration and canopy cooling^87^. Under limited soil moisture, the capacity for evaporative cooling is reduced, potentially exacerbating heat stress impacts on yield^12,81,88^. The weak relationships observed here between heat stress intensity and rapeseed yield therefore suggest that yield responses may be better explained by compound heat–water stress interactions rather than by temperature or rainfall metrics alone. Future studies could explicitly investigate these compound effects on UK rapeseed using multivariate or multiple regression approaches to better capture yield responses.

### Limitations and further work

Although this study analysed the heat and cold stresses on UK arable lands for rapeseed and their spatiotemporal trends, several limitations exist. Firstly, the temperature dataset used were not uniform between heat and cold stress. While daily *T*_*max*_ was used for estimations of heat stress, assessments of cold stress were based on *T*_*min*_ as this study wanted to understand the limiting magnitude of both temperature stresses on UK lands for rapeseed crop. This heterogeneity might overestimate the values of stresses caused by both high temperatures and low temperatures.

Secondly, GAEZ v4 attainable yield data do not represent actual agricultural yields, but rather provide a standardized spatial reference or normalization tool for assessing relative climate-driven production potential^55^. This study has primarily discussed temperature stress on UK lands as potential rapeseed cropping regions. Other climatic, edaphic and nutritional factors such as water availability, topography, fertilizers are important determinants of rapeseed productivity^22,38,89,90^. Similarly, although this study has focused on the temperature stress analysis on lands suitable for rapeseed, these areas included soils for horticulture and other crops, suggesting that the stress values estimated may not be relevant for those areas that were not used for crop cultivation^42^. Additionally, the stress analysis was modelled primarily on fixed arable land pixels throughout the study period, without considering land cover change driven by climate variability or agricultural practice^75,81^. The weak yield-heat stress relationships identified here further highlight that yield responses to warming cannot be fully understood without accounting for compound stress interactions and non-climatic drivers, particularly at regional scales. Future work can consider investigating additional climatic, agricultural and edaphic conditions on UK lands for rapeseed, alongside temperature stress.

Heat stress negatively impacts rapeseed yield components, such as the number of pods, especially during flowering and grain filling^29,91,92^. This study investigated potential yield loss due to heat stress using attainable yield data for UK rapeseed under constrained conditions^55^. However, analysing yield loss based on key components (e.g., seed number and weight) could provide deeper insights into the effects of climatic stresses on productivity^28,29^. Such analysis is challenging at a large scale due to the lack of yield component data. Spaceborne remote sensing, using indices like the Normalized Difference Yellowness Index (NDYI), could assess yield components (e.g., flower number) over large areas^28,64^. The information of flower numbers can facilitate estimating the number of pods or final yield based on agricultural knowledge or statistical models^29,64,93^. This would improve yield estimates and better quantify the impact of heat stress on rapeseed productivity. The weak *f*_*HS*_-yield regression relationships may also result from the limited yield data available (2016–2024), suggesting the need for further studies with extended datasets.

Finally, the temperature thresholds selected in the stress analyses were for generic rapeseed varieties commonly grown in the UK and global scale. However, the thresholds can differ by variety and growth stage^65^. Advances in breeding research and technology have enabled the development of cultivars which are tolerant to high and low temperatures or have development phases independent of temperature, which may mitigate against the trends in heat stress observed in this study^94^. Additionally, whilst this study employed intensity and frequency as stress metrics, incorporating stress duration can provide a more holistic assessment of temperature stress on crops including UK rapeseed^79–81^. Further work could also consider applying these indices to predictive climate model outputs under multiple SSP (Shared Socioeconomic Pathways) scenarios, incorporating phenology simulations to account for anticipated shifts in flowering and reproductive stages, thereby enabling assessment of threshold exceedances and climate-induced production risks for UK rapeseed in the coming decades^95,96^.

## Conclusion

The present study investigated the spatiotemporal patterns of heat stress and cold stress on UK arable lands for rapeseed cropping, based on 1 km × 1 km temperature data. Heat stress values and percentages of days experiencing the stress during flowering and reproductive phases increased from 1961 to 2020, showing a growing spatial pattern from the south to north of the UK. Cold stress values and percentages of days experiencing cold stress during flowering, vegetative and reproductive periods showed decreasing trends, especially in England. This work also revealed that heat stress was increasing at a faster rate than cold stress has been decreasing during the flowering period, though this result depends on the models and parameter values used, and alternative approaches could potentially show different relative trends. These results indicate that global warming has enhanced high temperature stress while reducing low temperature stress on UK rapeseed crops. The normalized rapeseed production loss index revealed an increasing risk, with significant difference between decades and UK regions, due to heat stress on UK rapeseed cropping from 1961 to 2020. Furthermore, while heat stress during flowering differentially (positively vs. negatively) and weakly (R^2^ ≤ 0.30) impacted reported rapeseed yields of UK regions over 2016–2024 (*p* > 0.05), higher intensities of heat stress would reduce crop yields. Despite its relatively slow rate of increase over the past six decades and that currently rapeseed yields appear to be resilient to heat stress, this study demonstrates a steadily increasing risk of heat stress for rapeseed in the UK particularly in regions with the largest rapeseed areas.

While this study provided insights on environmental stress on UK rapeseed, inclusion of other limiting factors such as topography and precipitation/soil moisture are expected to improve the understanding of abiotic stresses for rapeseed. Additionally, it is also interesting to know if the mild heat stress experienced by UK rapeseed crop was captured by spaceborne imagery. Given this, future work will investigate whether the estimated temperature stress on UK rapeseed crop is observable using remote sensing imagery. For sustainable rapeseed cultivation, it is also of importance for the UK to continue investing in mitigation and adaptation strategies such as crop land management and breeding schemes against climate variability.

## Supplementary Information

Below is the link to the electronic supplementary material.

Supplementary Material 1

## Data Availability

All used datasets in this study are publicly available. The temperature dataset used could be accessed from https://data.ceda.ac.uk/badc/ukmo-hadobs/data/insitu/MOHC/HadOBS/HadUK-Grid/v1.2.0.ceda/1km^53^. Rapeseed attainable yield (Yattainable) in global grids can be accessed from GAEZ v4 portal: https://gaez.fao.org/pages/data-access-download^55^. The crop suitability index in classes for rapeseed for current cropland in grid cell of the period 1961-1990 was obtained from the website https://gaez-services.fao.org/apps/theme-4/. The share of cultivated land can be accessed from GAEZ v3 platform (https://www.gaez.iiasa.ac.at/)^56^. The shapefiles of the UK nations and England regions can be obtained from: https://geoportal.statistics.gov.uk/^40,41^. Land cover map 1990 of the UK can be retrieved from Digimap at https://digimap.edina.ac.uk/^42^. Other data will be made available from the corresponding author upon reasonable request.
