# Supplementary material for: Spatiotemporal dynamics of heat stress and cold stress on UK rapeseed cropping over 1961–2020
Source: Sci Rep. 2026 Mar 5;16:12263. doi: 10.1038/s41598-026-41957-7 (PMC13079783; doi:10.1038/s41598-026-41957-7)
Supplement: Supplementary file 1 — Supplementary Material 1 [file 41598_2026_41957_MOESM1_ESM.docx]

Supporting information for

**Spatiotemporal dynamics of heat stress and cold stress on UK rapeseed cropping over 1961-2020**

Biao Hu^*,a^ , Mark E. J. Cutler^a^, Alexandra C. Morel^*,a^

^a^ Division of Energy, Environment & Society, School of Humanities, Social Sciences and Law, University of Dundee, Nethergate, Dundee DD1 4HN, UK

*Corresponding authors, bh46824@foxmail.com; [amorel001@dundee.ac.uk](mailto:amorel001@dundee.ac.uk)


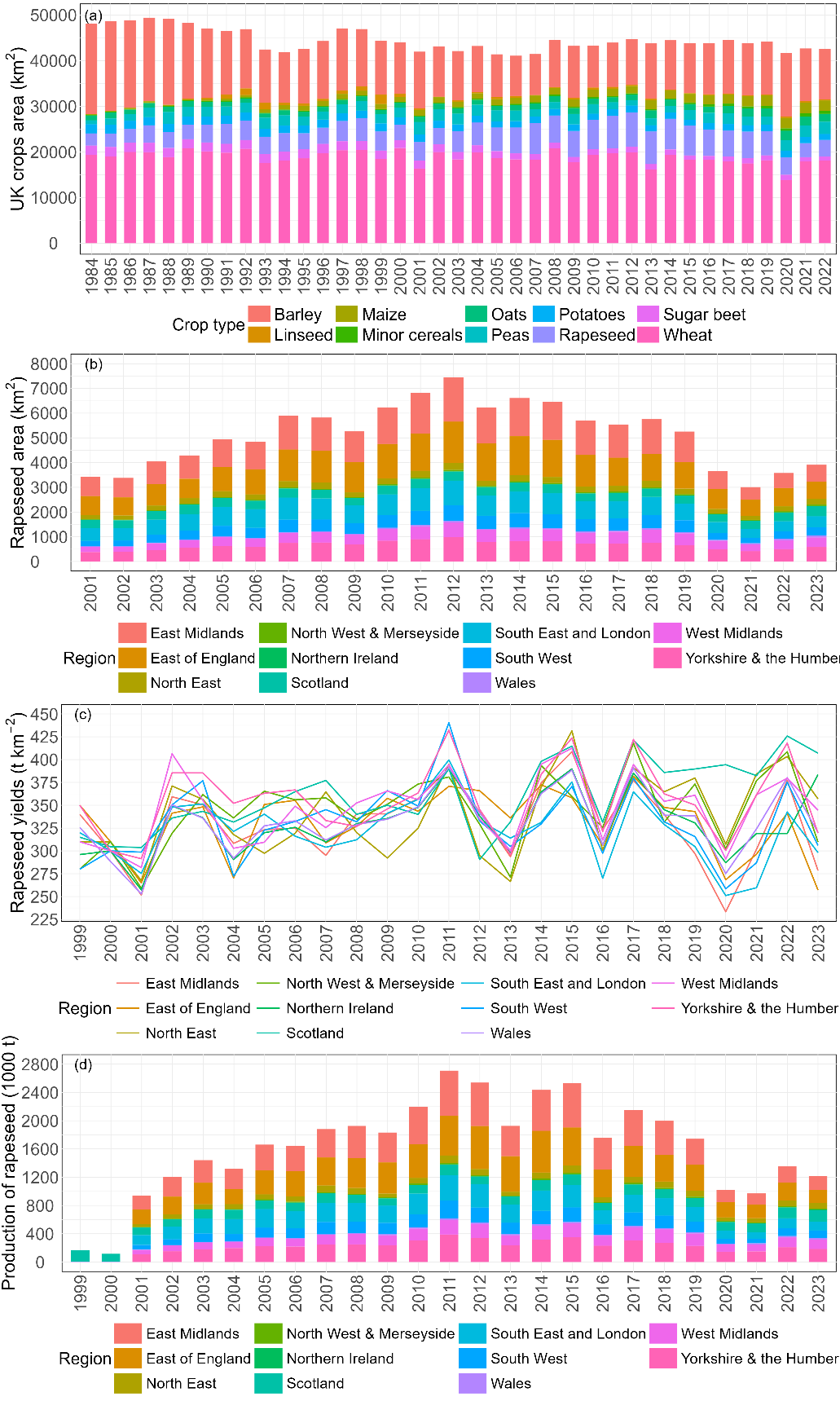


**Fig.S1:** (a) Cultivation area of rapeseed and other main crops of the UK from 1984 to 2022, and cultivation area (b) of rapeseed for UK regions from 1999 to 2023. Source: data were obtained from[[1](#_ENREF_1)]. For data of regions in England, the values are for winter sown rapeseed.

**Fig.S2** shows the principal growth stages (GS0: Germination and emergence; GS1: Leaf development; GS2: Side-shoot formation; GS3: Stem elongation/extension; GS4: Does not apply to oilseed rape; GS5: Inflorescence/flower-bud emergence; GS6: Flowering; GS7: Pod/seed (fruit) development; GS8: Pod/seed (fruit) ripening; GS9: Senescence) of rapeseed in the UK based on the Biologische Bundesanstalt, Bundessortenamt und CHemical Industry (BBCH) system[[2](#_ENREF_2)].


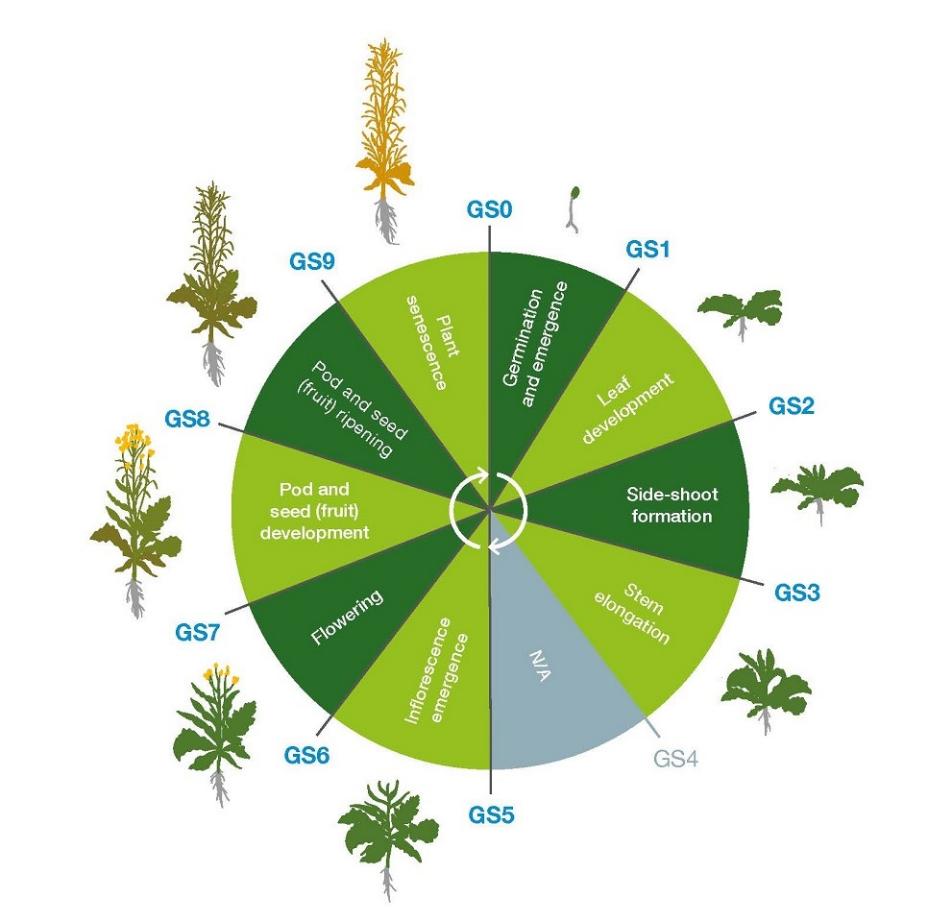


**Fig.S2**. Principal growth stages of rapeseed crop in the UK [[2](#_ENREF_2)].

In **Fig.S3,** the crop (i.e., rapeseed) attainable yield (*Y_attainable_*) in global grids was obtained from the site (https://gaez.fao.org/pages/data-access-download) published by FAO for Global Agro-Ecological Zones v4 (GAEZ v4)[[3](#_ENREF_3)] and the share of cultivated land at the global scale was downloaded from the site (<https://www.gaez.iiasa.ac.at/>) provided by Global Agro-Ecological Zones v3 (GAEZ v3)[[4](#_ENREF_4)]. Both the attainable yield and the share of cultivated land data were clipped to the UK landscape and resampled to 1 km resolution.


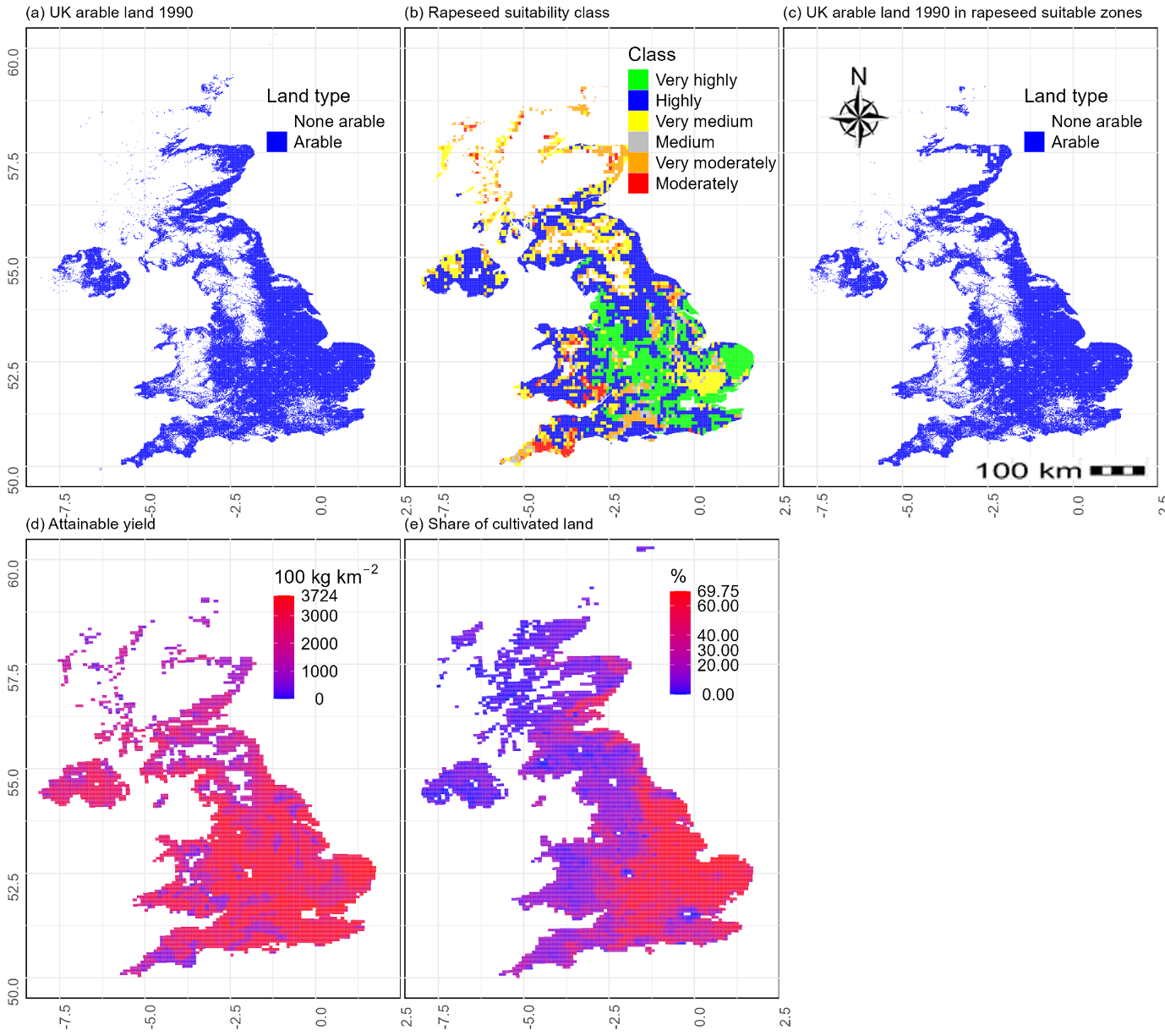


**Fig.S3**: (a) UK arable and horticulture land 1990[[5](#_ENREF_5)]; (b) Land suitability classes for rapeseed crop in the UK; (c) UK arable land pixels in rapeseed suitability zones ; (d) Average attainable yield of rapeseed (current cropland, the time period 1961-1990, rainfed conditions, high input level, with CO2 fertilization, climate data source CRUTS32 based on historical data for the time period 1981-2010) for current cropland in the UK; (e) The share of land that is under cultivation for the UK in the year 2000.

The most newly Global Agro-Ecological Zones v4.0 (GAEZ v4) covers six themes include (a) land and water resources, (b) agro-climatic resources, (c) agro-climatic potential yield, (d) suitability and attainable yield, (e) actual yields and production, and (f) yield and production gaps[[3](#_ENREF_3)]. Theme four offers information for 53 crops (rapeseed, etc.) and has sub-themes on suitability class, suitability index, agro-ecological attainable yield, and crop water indicators (<https://gaez.fao.org/pages/theme-details-theme-4>). The suitability index is defined as: SI = 100 * (90 * VS + 70 * S + 50 * MS + 30 * mS +15 * VmS+ 0 * NS)/0.9; with values between 0 and 10000 and where VS, S, …, NS are the area shares of different suitability classes in a 5 arc-minute grid cell (<https://gaez.fao.org/pages/theme-details-theme-4>). The suitability class is comprehensively estimated from input data including water supply, climate models, time period, representative concentration pathways (RCPs), input (high or low) levels, on/off of CO_2_ fertilization. The suitability class (**Tab.S1**) in gridded map could be also integrated with crop packages, which can produce maps containing cultivation locations per suitability class of crops studied.

**Tab.S1**: Suitability class of rapeseed adapted from GAEZ v4 dataset.

| **Suitability class** | **Suitability description** | **Farm economics[**[3](#_ENREF_3)**]** |
| --- | --- | --- |
| S1 | very highly suitable land (>90% of maximum attainable yield) | Prime land offering best conditions for economic crop production |
| S2 | highly suitable land (80-90% of maximum attainable yield) |  |
| S3 | very medium suitable land (70-80% of maximum attainable yield) | Good land for economic crop production |
| S4 | medium suitable land (60-70% of maximum attainable yield) |  |
| S5 | very moderately suitable land (50-60% of maximum attainable yield) | Moderate land with substantial climate and/or soil/terrain constraints requiring high product prices for profitability |
| S6 | moderately suitable land (40-50% of maximum attainable yield) |  |
| S7 | very marginally Suitable land (30-40% of maximum attainable yield) | Commercial production not viable. Land could be used for subsistence production when no other land is available |
| S8 | marginally suitable land (20-30% of maximum attainable yield) |  |
| S9 | Not suitable (<20% of maximum attainable yield) | Economic production not feasible, and not suitable (0% of maximum attainable yield) |

**
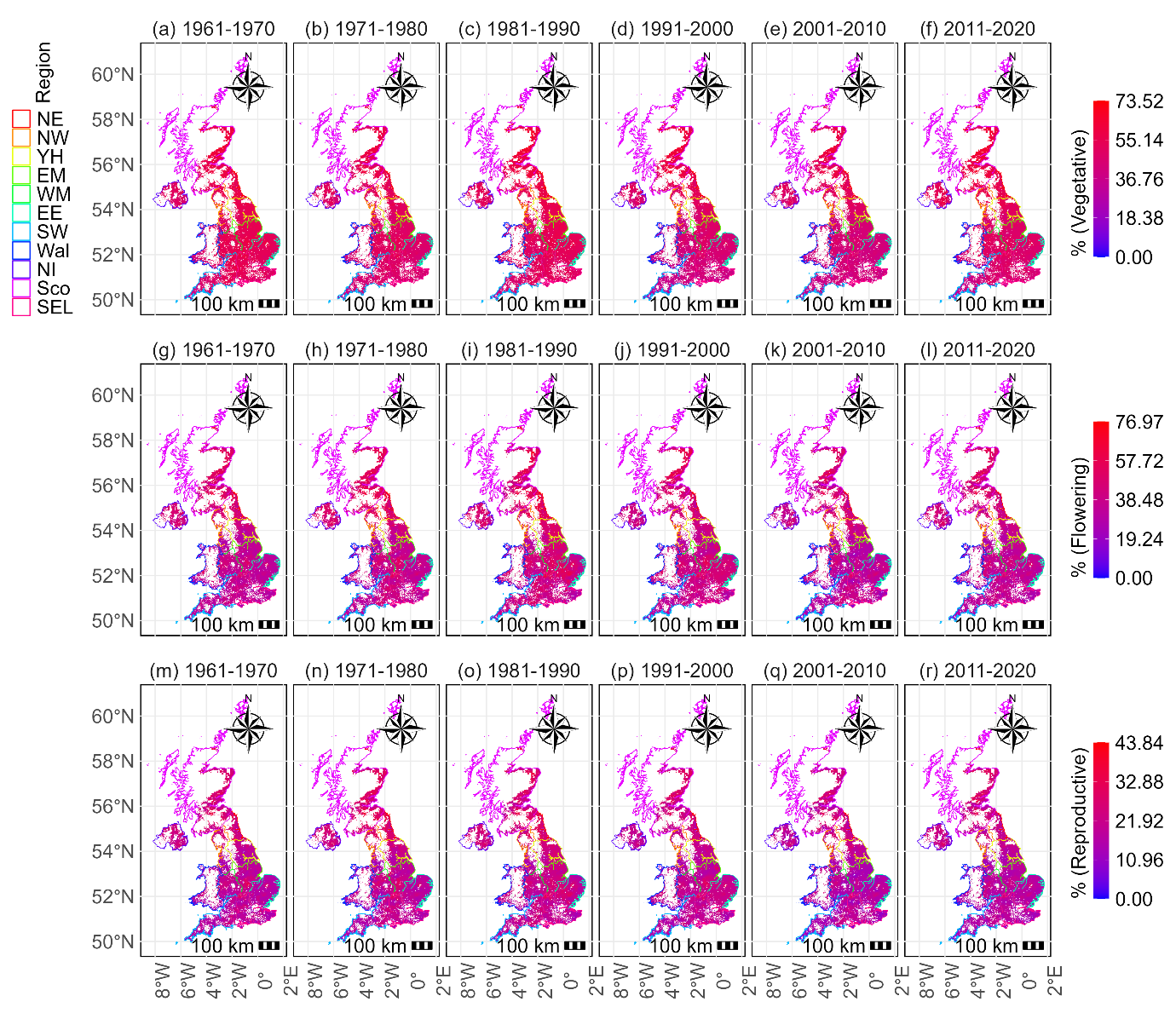
**

**Fig.S4**: Maximum percentages of days experiencing cold stress of UK arable lands for rapeseed cropping during (a-f) vegetative, (g-l) flowering and (m-r) reproductive stages of each decade during 1961-2020. NE indicates North East, NW indicates North West, YH indicates Yorkshire and The Humber, EM indicates East Midlands, WM indicates West Midlands, EE indicates East of England, SW indicates South West, Wal indicates Wales, NI indicates Northern Ireland, Sco indicates Scotland, SEL indicates South East and London. The same letters are used in the figures showing spatial patterns in this study.


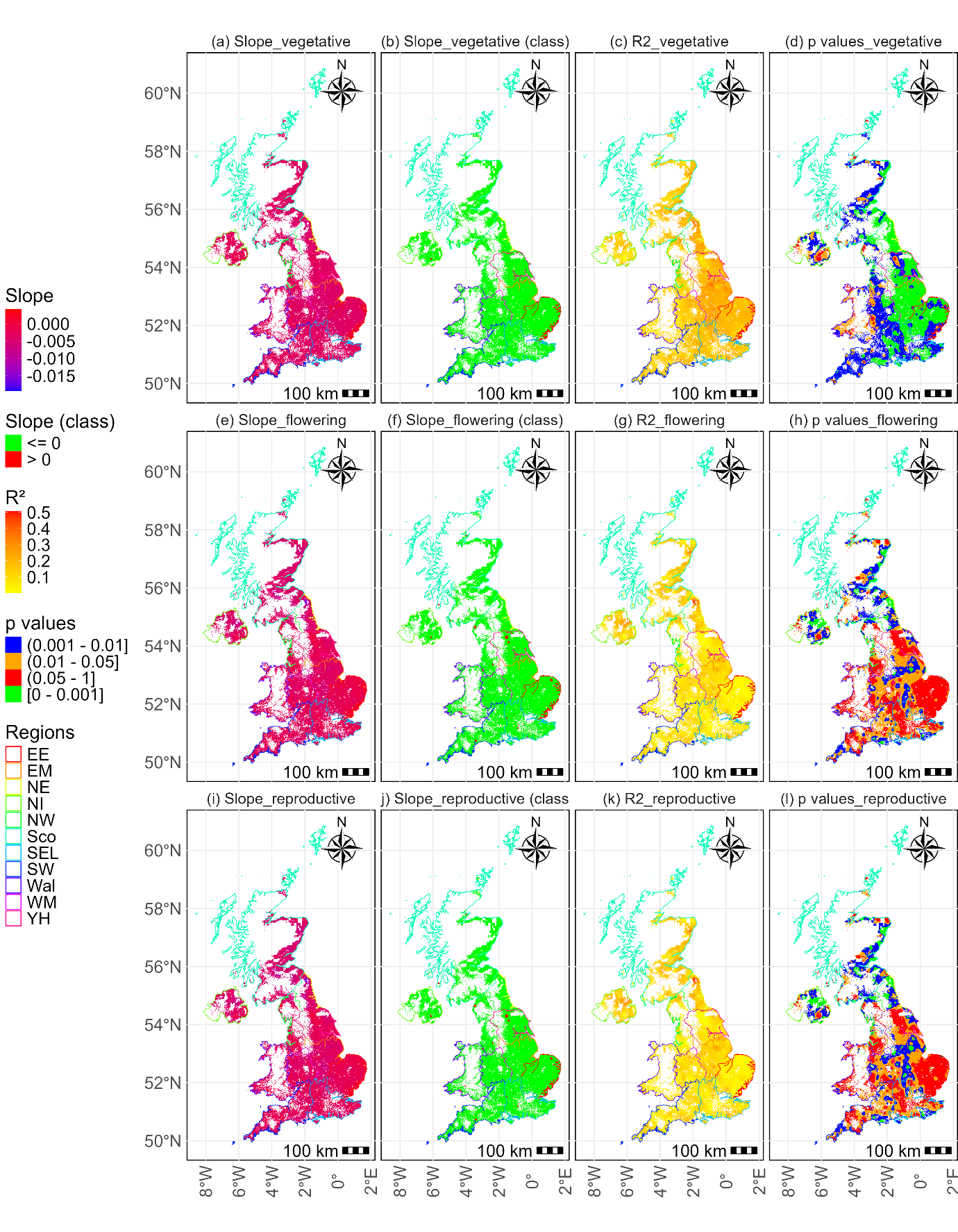


**Fig.S5**: Pixel-wise map of trend, trend (in class), R^2^ and p values of annual *CDD* on UK arable lands for rapeseed cropping during vegetative, flowering and reproductive stages from 1961 to 2020.

**Fig.S6** was obtained as the difference between annual mean *T_min_* of December of the UK arable land from 1960 to 2020 and the mean *T_min_* (1.79°C) of December of 1980-2010.


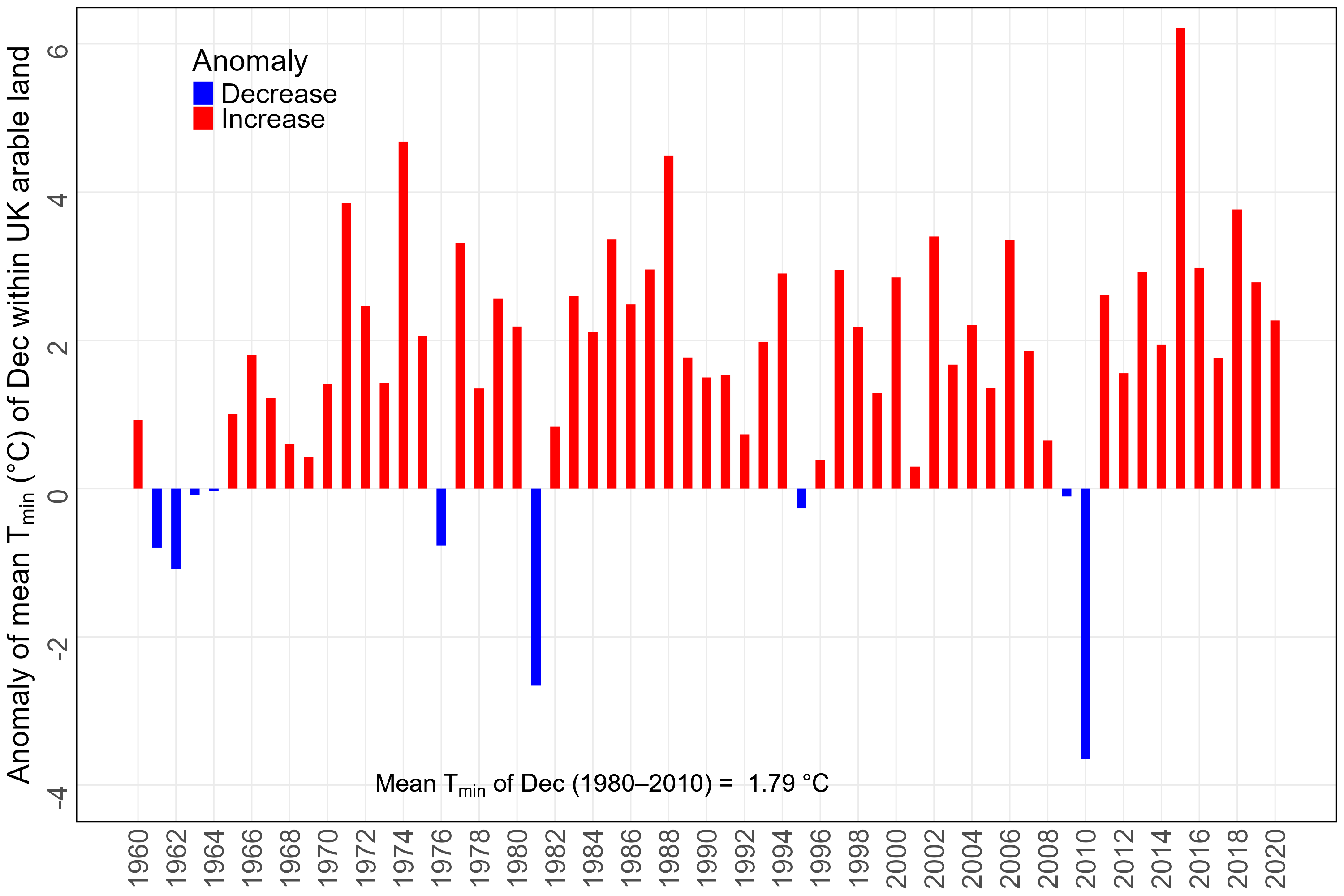


**Fig.S6:** Anomaly of mean T_min_ (°C) of December on arable land of the UK from 1960 to 2020.


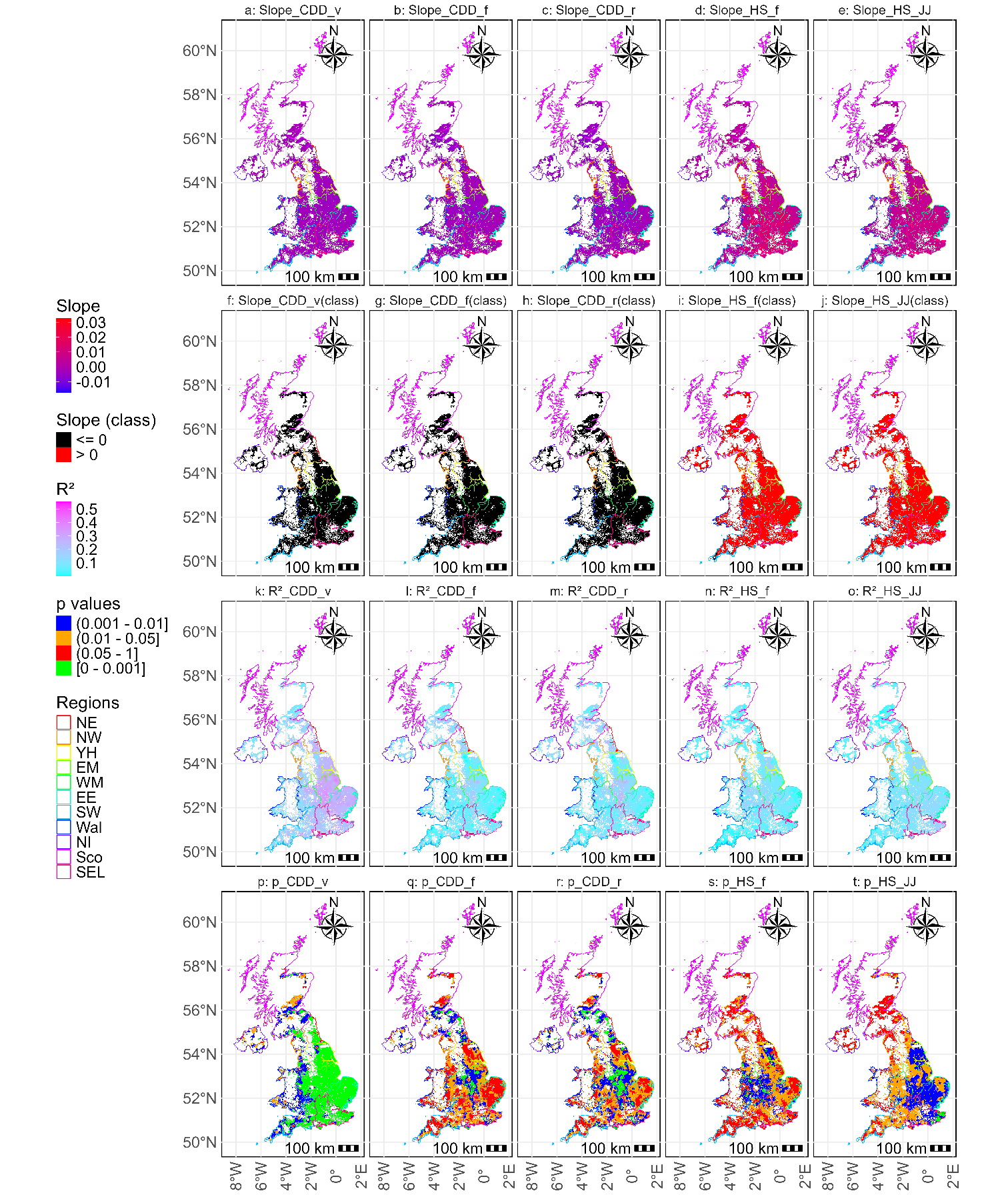


**Fig.S7:** Pixel-wise map of trend, trend (in class), R^2^ and p values of annual percentages of days experiencing cold stress (marked with *CDD*) and heat stress (marked with *HS*) on UK arable lands for rapeseed cropping during vegetative, flowering and reproductive (for cold stress), flowering and June and July (for heat stress) from 1961 to 2020. The ‘v’ ‘f’, ‘r’ and ‘JJ’ indicate vegetative, flowering, reproductive, and June and July, respectively.


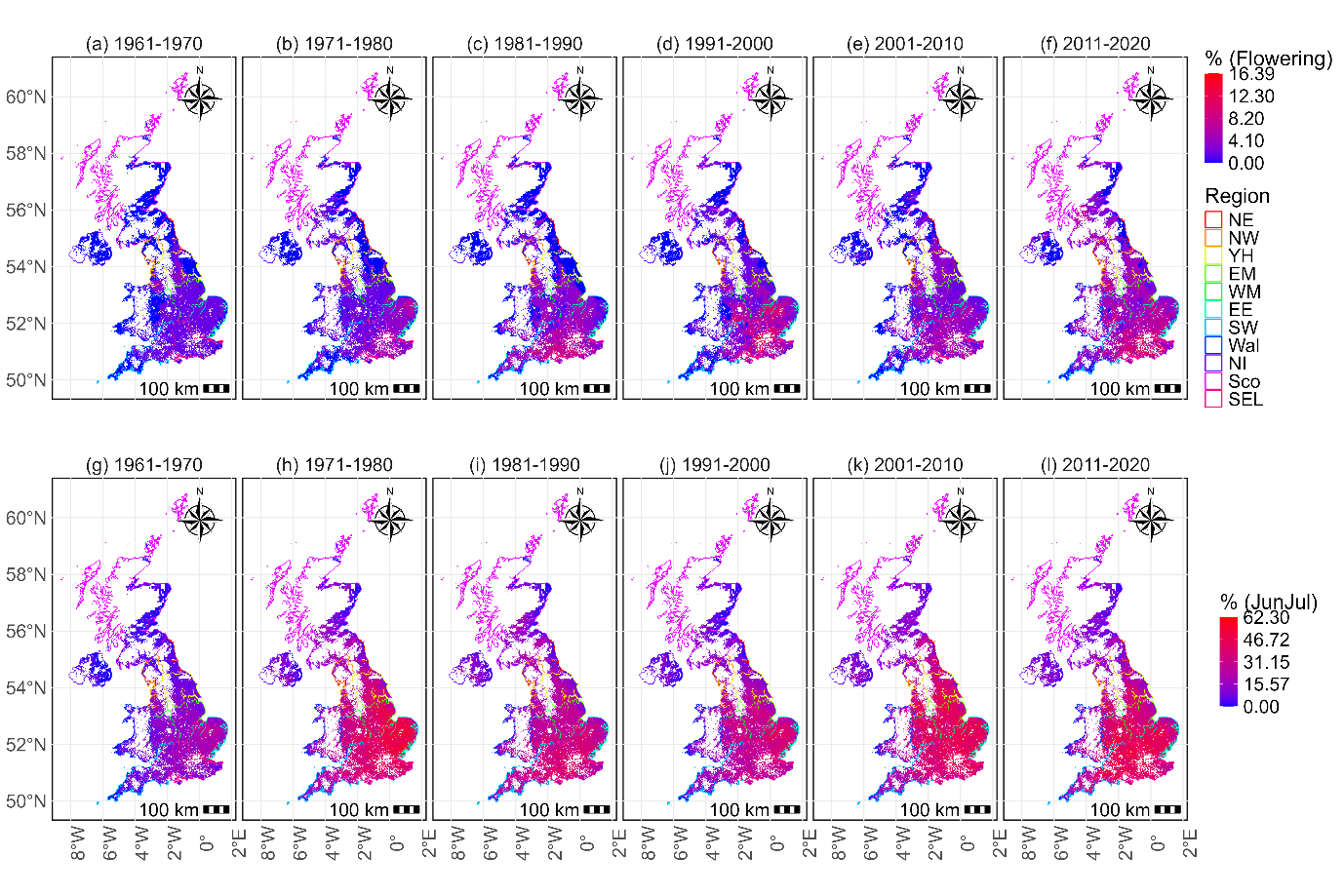


**Fig.S8**: Maximum percentages of days experiencing heat stress of UK arable lands for rapeseed cropping during (a-f) flowering stage and (g-l) June and July.


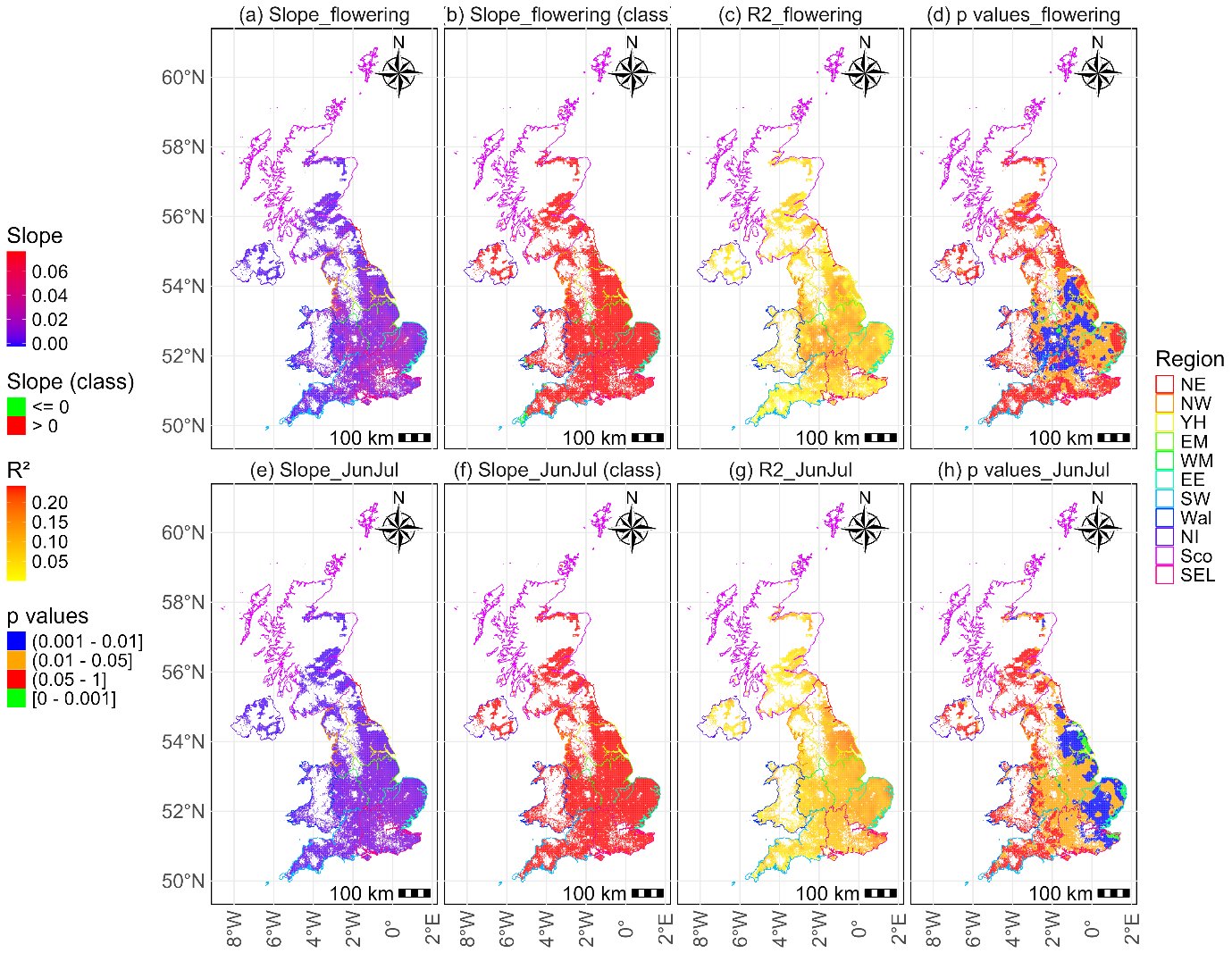


**Fig.S9:** Pixel-wise map of trend, trend (in class), R^2^ and p values of annual *f_HS_* on UK arable lands for rapeseed cropping during flowering stage, and June and July from 1961 to 2020.

**Tab.S2:** Decadal cold stress values of UK arable lands for rapeseed cropping for its vegetative and reproductive periods from 1961 to 2020.

| Periods | 1961-1970 | 1971-1980 | 1981-1990 | 1991-2000 | 2001-2010 | 2011-2020 |
| --- | --- | --- | --- | --- | --- | --- |
| CDD value (min-max); vegetative | 134.48 - 986.59 | 108.78 - 882.04 | 94.81 - 926.48 | 64.11 - 844.03 | 88.36 - 738.6 | 67.32 - 804.35 |
| CDD value (mean±sd); vegetative | 599.62 ± 92.87 | 452.4 ± 78.28 | 455.07 ± 84.65 | 312.08 ± 70.28 | 327.99 ± 73.11 | 348.82 ± 76.85 |
| Percentage (%) of days experiencing cold stress (min-max); vegetative | 26.17 - 73.85 | 20.75 - 74.48 | 18.38 - 78.26 | 15.15 - 79.76 | 19.34 - 71.79 | 13.68 - 74.27 |
| Percentage (%) of days experiencing cold stress (mean±sd); vegetative | 52.4 ± 4.86 | 48.19 ± 4.95 | 51.95 ± 5.55 | 44.19 ± 7.25 | 43.26 ± 6.27 | 47.85 ± 6.73 |
| CDD value (min-max); reproductive | 6.92 - 216.11 | 9.18 - 238.99 | 6.46 - 201.83 | 4.75 - 188.77 | 4.22 - 180.48 | 8.45 - 207.22 |
| CDD value (mean±sd); reproductive | 67.24 ± 20.18 | 57.36 ± 19.48 | 64.65 ± 21.03 | 62.72 ± 20.41 | 42.31 ± 16.09 | 57.43 ± 16.56 |
| Percentage (%) of days experiencing cold stress (min-max); reproductive | 5.96 - 51.62 | 6.59 - 56.43 | 5.74 - 47.77 | 3.65 - 49.82 | 3.28 - 43.39 | 4.92 - 51.29 |
| Percentage (%) of days experiencing cold stress (mean±sd); reproductive | 19.45 ± 4.71 | 20.62 ± 5.59 | 21.79 ± 4.88 | 20.16 ± 4.45 | 17.43 ± 4.85 | 18.13 ± 4.51 |
| min: minimum; max: maximum; sd: standard deviation. The unit of CDD: ℃ d yr^-1^ | | | | | | |

The *R^2^* reflects the degree of fit between the predicted and observed values of normalized production loss index.


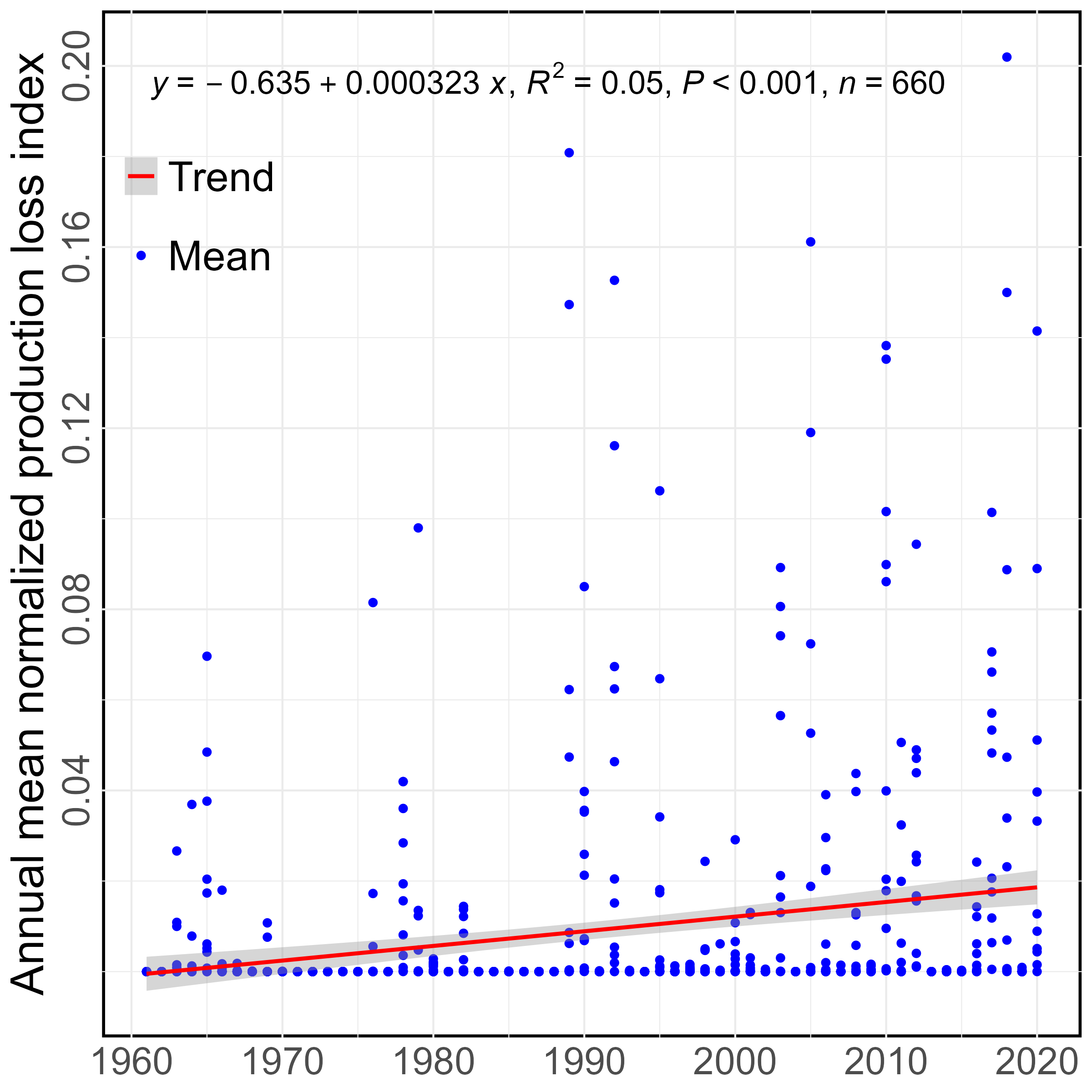


**Fig.S10**: Annual mean values of normalized production loss index (f_RPL_nm_) in rapeseed of the UK regions due to heat stress in flowering period between 1961 and 2020 and their trendline. Index values were normalized by the maximum annual f_RPL_ of each region each year.

**References**

1. Defra, Cereal and oilseed rape production [Accessed on 09/02/2025]. 2024.

2. AHDB, Oilseed rape growth guide [Accessed 30/07/2023]. 2023.

3. Fischer, G., et al., Global Agro-Ecological Zones v4 – Model documentation. Rome, FAO. 2021.

4. IIASA/FAO, Global Agro‐ecological Zones (GAEZ v3.0). IIASA, Laxenburg, Austria and FAO, Rome, Italy. 2012.

5. EDINA, Land Cover Map 1990 [FileGeoDatabase geospatial data], Scale 1:250000, Tiles: GB, Updated: 1 December 1990, CEH, Using: EDINA Environment Digimap Service, <<https://digimap.edina.ac.uk>>, Downloaded: 2023-11-09 09:30:39.451. 1990.
